# The network-based underpinnings of persisting symptoms after concussion: a multimodal neuroimaging meta-analysis

**DOI:** 10.1038/s44220-025-00503-6

**Published:** 2025-09-23

**Authors:** Adriano Mollica, Robin F. H. Cash, Carl Froilan D. Leochico, Peter Giacobbe, Isabella J. Sewell, Andrew Zalesky, Jennifer S. Rabin, Maged Goubran, Simon J. Graham, Benjamin Davidson, Fa-Hsuan Lin, Nir Lipsman, Clement Hamani, Matthew J. Burke, Sean M. Nestor

**Affiliations:** 1 Harquail Centre for Neuromodulation and Hurvitz Brain Sciences Program, Sunnybrook Research Institute, Toronto, Ontario Canada; 2 Department of Psychiatry, Sunnybrook Health Sciences Centre, University of Toronto, Toronto, Ontario Canada; 3 Systems Group, Department of Psychiatry, University of Melbourne, Melbourne, Victoria Australia; 4 Biomedical Engineering, University of Melbourne, Melbourne, Victoria Australia; 5 Department of Physical Medicine and Rehabilitation, St Luke’s Medical Center, Bonifacio Global City, Philippines; 6 Toronto Rehabilitation Institute, University Health Network, Toronto, Ontario Canada; 7 Rehabilitation Sciences Institute, University of Toronto, Toronto, Ontario Canada; 8 Physical Sciences Platform and Hurvitz Brain Sciences Program, Sunnybrook Research Institute, Toronto, Ontario Canada; 9 Department of Medical Biophysics, University of Toronto Faculty of Medicine, Toronto, Ontario Canada; 10 Division of Neurosurgery, Sunnybrook Health Sciences Centre, University of Toronto, Toronto, Ontario Canada; 11 Division of Neurology, Department of Medicine, Sunnybrook Health Sciences Centre, University of Toronto, Toronto, Ontario Canada

**Keywords:** Brain injuries, Translational research

## Abstract

Persisting symptoms after concussion (PSaC) represent a complex and poorly understood neuropsychiatric phenomenon with limited treatment options. Neural network dysfunction has been associated with PSaC, and neuromodulation, particularly repetitive transcranial magnetic stimulation, may be a promising intervention. However, neuroimaging findings have been inconsistent, limiting understanding of underlying network dysfunction. We aimed to identify a core neural network associated with PSaC and explore whether this network could yield candidate cortical targets for neuromodulation at the individual level. We hypothesized that differences in network disruption would be evident between individuals with high versus low symptom burden in PSaC. Here we show that a convergent multi-analytic approach combining symptom–activation maps generated from existing fMRI datasets, systematic review of resting-state fMRI studies of PSaC, and network-based meta-analysis of coordinates derived from these studies co-localize to the salience network in high symptom burden PSaC. Using Human Connectome Project data, we mapped this network to cortical regions that could serve as individualized targets for neuromodulation. This aligns with current clinical models of PSaC and may present a new direction for network-based therapy.

## Abstract (web summary)

Persisting symptoms after concussion (PSaC) present a complex neuropsychiatric challenge with limited treatment options due to inconsistent neuroimaging findings. Here the authors employ a multi-analytic approach to identify the salience network as a core dysfunction hub in PSaC, proposing specific cortical regions as potential targets for personalized neuromodulation therapies.

## Main

Persisting symptoms after concussion (PSaC) (formerly referred to as post-concussive syndrome) represents a complex neuropsychiatric phenomenon that occurs in 10–30% of patients^1^. It is characterized by a disparate range of physical, cognitive and emotional symptoms, persisting for at least 4 weeks beyond the acute phase of injury^2,3^. The polysymptomatic nature of PSaC and lack of objective biomarkers necessitate a multipronged, symptom-targeted treatment approach^4^. Current clinical guidelines emphasize a multidisciplinary framework that includes individualized management of symptoms such as headache, depression, vestibular dysfunction, cognitive impairment and fatigue, often involving a combination of physical therapy, cognitive rehabilitation, pharmacological treatments and psychological interventions^5,6^. Within PSaC, there is substantial heterogeneity regarding the degree of neurological insult, mechanism of injury, symptom presentation, duration and perceived severity, as well as predisposing and perpetuating biopsychosocial factors^5,7,8^. Key risk factors for a high burden of persisting symptoms include female sex, non-white ethnic groups, substance use, pre-existing psychiatric conditions, history of chronic pain and history of somatization. These sources of variability challenge the notion of a uniform pathophysiological mechanism to explain PSaC^4,9–11^. However, the answer to why some individuals develop persisting symptoms while others recover may be rooted in large-scale neural network dysfunction^12–14^. Advances in neuroimaging and image-guided neuromodulation offer new avenues for measuring and targeting these network dysfunctions, which could be a meaningful addition to the toolbox of management strategies for PSaC^15,16^.

Symptom perpetuation in PSaC has been linked to heightened vigilance and attentional bias toward bodily sensations, negative attributions and fear-driven avoidance^17–19^. At a neural level, these processes are probably mediated by dysfunction in large-scale networks involved in sensory processing, attentional control and cognitive–emotional integration, particularly the salience network, executive-control network, default-mode network and somatomotor network^20–24^. Disruptions across these networks have been associated with PSaC, with altered connectivity patterns linked to symptom severity, impaired cognitive control and emotional dysregulation^23,25,26^. Heightened interactions between sensory processing regions and attentional networks suggest a maladaptive reinforcement of symptom perception^14,27^, while increased connectivity within cognitive control networks may represent compensatory mechanisms that ultimately contribute to inefficiencies and cognitive symptoms^22,25^. Despite these insights, it remains unclear whether dysfunction in a specific network plays a dominant role in driving the polysymptomatic presentations associated with PSaC^12,20^. Given the potential for network-based interventions, such as neuromodulation, a clearer understanding of the neural correlates underlying PSaC could provide critical insights for targeted treatment strategies^15^.

Neuromodulation approaches that directly target functional brain networks, such as repetitive transcranial magnetic stimulation (rTMS), have demonstrated mixed results for treating PSaC^28,29^. Two studies have evaluated the effects of left dorsolateral prefrontal cortex (DLPFC) rTMS on global symptoms of PSaC^30,31^. While one open-label pilot study reported a large effect size (Cohen’s *d* = 0.91)^30^, a double-blind sham-controlled trial found no therapeutic benefit compared with sham stimulation^31^. These previous rTMS studies targeted the DLPFC due to its central role in cognitive control networks and its established use in depression treatment^28,32^. However, depression accounts for a substantial portion of the experience of PSaC and shares overlapping neural mechanisms, making it difficult to disentangle the effects of DLPFC rTMS on mood from its potential impact on other PSaC-related symptoms^33–36^. This challenge underscores the need for a more mechanistically driven approach that accounts for individual network dysfunction rather than relying on anatomical targets derived from other conditions^16,28^. Establishing a clearer understanding of the neural mechanisms underlying PSaC may help optimize network-based neuromodulation strategies and expand treatment options.

**Fig. 1:**
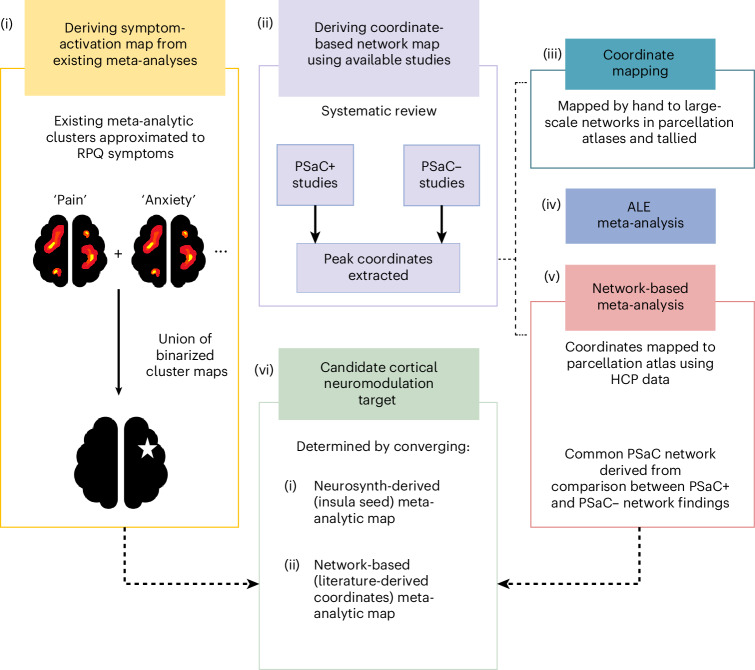
Flow diagram of neuroimaging analyses. A converging multi-analytical approach was used to explore network dysfunction in PSaC that included (i) generation of symptom–activation maps from existing fMRI datasets available on Neurosynth to identify common areas of activation across PSaC symptoms; (ii) systematic review of PsaC neuroimaging studies to contrast network alterations in those with high versus low symptom burden after concussion; (iii) mapping and tallying extracted coordinates individually to large-scale networks in parcellation atlases; (iv) Activation Likelihood Estimation (ALE) meta-analysis of included studies using a seed-based connectivity approach; (v) network-based meta-analysis of coordinates derived from whole-brain connectivity to contrast high versus low symptom burden patterns of altered connectivity; and (vi) determination of a functional cortical signature for neuromodulation targeting with Human Connectome Project (HCP) data.

In this study, we aimed to (1) identify a core neural network from functional magnetic resonance imaging (fMRI) associated with PSaC and (2) explore whether this network could yield candidate cortical targets for neuromodulation at the individual level. We hypothesized that differences in network disruption would be evident between individuals with high versus low symptom burden in PSaC. High versus low symptom burden was determined on the basis of the primary clinical outcome measures used in each study, including predefined cut-off scores, categorical distinctions (for example, severity ratings of specific symptoms) or, when a clear cut-off score was not defined, classification was based on whether the total score at the time of neuroimaging assessment was above or below recognized thresholds for the measure being used. Leveraging a multi-faceted set of neuroimaging analyses across different data sources (Fig. 1), we identified the network-based underpinnings of PSaC and localized an optimal candidate target for neuromodulation therapies.

## Results

### PSaC symptom-based network mapping

**Fig. 2:**
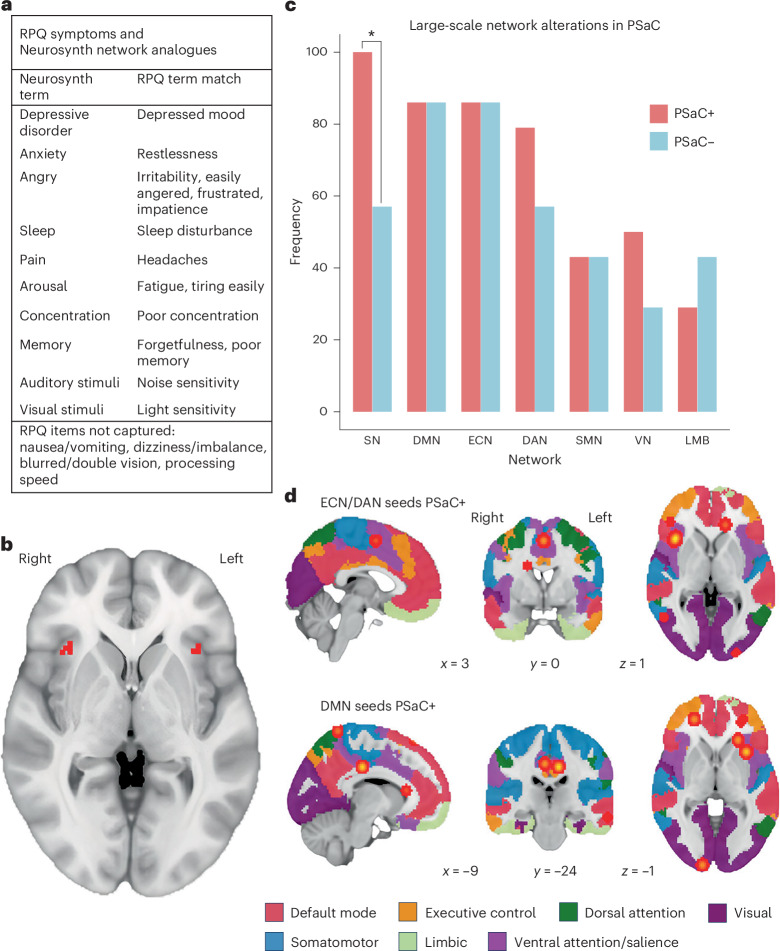
PSaC symptom-based map and coordinate-based analyses. **a**, RPQ symptoms and Neurosynth functional network analogs. **b**, Symptom–activation map derived from Neurosynth data, showing the union of 11 RPQ-derived terms with bilateral insula activation. **c**, Alterations across large-scale networks in PSaC+ versus PSaC– studies determined by network mapping on parcellation atlases. The only significant difference found between groups was in the salience network (*P = 0.026, Fisher’s exact test). **d**, Unthresholded and non-significant ALE image results of activations/increased connectivity between seeds in the executive-control network (ECN)/dorsal attention network (DAN) (top panel), default-mode network (DMN) (bottom panel), and peak regions in PSaC+ studies. Cross sections provided are core regions of the salience network (bilateral insula and dorsal anterior cingulate cortex). Each image is overlaid on the MNI 152 brain with Yeo 2011 7-network parcellation atlas. LMB, limbic network; SMN, somatomotor network; SN, salience network; VN, visual network.

To identify a functional network clinically relevant to PSaC, we approximated 11 symptoms from the Rivermead Post-Concussion Questionnaire (RPQ) to available homologous terms in the Neurosynth database and extracted their corresponding meta-analytic activation maps (Fig. 2a). The union of 11 RPQ symptom–activation maps revealed an overlapping activation pattern that co-localized specifically to salience network topology, which was anchored by the right insula (Montreal Neurological Institute (MNI) coordinates 34, 21, 0) and left insula (MNI coordinates –35, 20, 0; Fig. 2b). As the Neurosynth-derived symptom maps did not primarily include studies of traumatic brain injury (TBI) or concussion patients, we conducted a separate coordinate-based meta-analysis using Neurosynth Compose, filtering for studies specifically involving participants with concussion (*n* = 37). The resulting activation map was binarized and multiplied with the RPQ symptom–activation map, which revealed consistent localization to the bilateral anterior insula (Supplementary Fig. 1).

### Systematic review of PSaC resting-state-based network mapping

To further investigate the neural networks implicated in PSaC, we conducted a systematic review of neuroimaging studies using resting-state fMRI that provided MNI coordinates for analysis. In addition to identifying common network alterations associated with PSaC, we sought to determine whether these patterns differed between individuals with high symptom burden (PSaC+) and those with low symptom burden or who had recovered (PSaC−).

A Preferred Reporting Items for Systematic Reviews and Meta-Analyses (PRISMA) flow diagram is presented in Supplementary Fig. 2. In total, 19 studies consisting of 1,300 unique participants met eligibility criteria. Group-wise demographic details, pertinent results and neuroimaging details are reported in Table 1 and Supplementary Tables 1 and 2, respectively. The proportion of female participants was 46.5% in PSaC+ studies, 45.4% in PSaC– studies and 26% in the two studies that included both groups. The mean and median time between injury and neuroimaging assessment was 28.3 and 10.2 months for PSaC+ studies and 6.6 and 5.0 months for PSaC– studies. Six out of 12 PSaC+ studies and 2 out of 5 PSaC– studies (as well as both studies comparing high and low symptom burden) excluded psychiatric conditions. Only 3 out of 12 PSaC+ studies and 1 out of 5 PSaC– studies (along with both high versus low symptom burden studies) excluded participants with a previous history of concussion (Supplementary Table 3).

**Table 1:** Characteristics of included studies

Study	Design	Sample size (mTBI, healthy controls)	Percentage female	Nature of mTBI	Mean symptom duration (months)	Primary symptom domain	Diagnostic approach to PSaC	Primary clinical outcome measure	Cut-off score	Clinician rated or self-rated
**PCS**+ **studies**										
Amir 2021 (ref. ^40^)	Cross-sectional	53 (27, 26)	70	Civilian	8.8	Global	Cut-off score	RPQ	RPQ ≥ 30	Self-rated
Dumkrieger 2019 (ref. ^37^)	Case-control	80 (44, 36)	36	Mixed	108	Headache	Headache specialist assessment	Numeric rating scale	ICDH-3 diagnostic criteria	Clinician rated
Leung 2016 (ref. ^41^)	Cross-sectional	30 (15, 15)	26	Veteran	70	Headache	Headache specialist assessment	Numeric rating scale, visual analog scale	ICDH-2 diagnostic criteria + visual analog scale score ≥ 3 out of 10	Clinician + self-rated
Rockswold 2019 (ref. ^42^)	Longitudinal	19 (10 PCS+ and 9 PCS-)	53	Civilian	3	Vision	Vision testing by optometrist	Optometric testing	Determined to have substantial oculomotor dysfunction	Clinician rated
Runyan 2022 (ref. ^44^)	Cross-sectional	127 (46, 81)	7	Veteran	11.6	Cognition	Cut-off score	NSI	Score of ≥3 on any NSI cognitive symptoms	Clinician rated
Shafi 2020 (ref. ^27^)	Cross-sectional	111 (80, 31)	33	Civilian	19	Global	Diagnostic assessment	DSM-IV PCS diagnostic criteria	DSM-IV PCS diagnostic criteria	Clinician rated
Sheth 2021 (ref. ^43^)	Cross-sectional	74 (49, 25)	10	Veteran	144	Post-traumatic stress disorder	Cut-off score	Clinician-Administered Post-traumatic Stress Disorder Scale	CAPS cut-off (score of ≥2)	Self-rated
Sours 2015 (ref. ^45^)	Longitudinal	56 (28, 28)	33	Civilian	6	Global	Cut-off score	RPQ	≥3 RPQ symptoms present at 3 months	Self-rated
Stevens 2012 (ref. ^46^)	Cross-sectional	60 (30, 30)	33	Civilian	2	Global	Total score, no cut-off^a^	Post-concussion symptoms checklist	No cut-off used; scores well above recognized threshold	Self-rated
Trofimova 2021 (ref. ^47^)	Longitudinal	22 (12, 10)	92	Mixed (civilian + sports)	2	Vestibular	Total score, no cut-off^a^	Post-Concussion Symptom Scale + Vestibular/Ocular Motor Screen	No cut-off used; scores well above recognized threshold	Clinician + self-rated
Vedaei 2023 (ref. ^38^)	Cross-sectional	100 (60, 40)	62	Civilian	24	Global	Diagnostic assessment	ICD-10 PCS diagnostic criteria	ICD-10 PCS diagnostic criteria	Clinician rated
Wong 2023 (ref. ^26^)	Cross-sectional	38 (17, 21)	100	Civilian	21.5	Global	Total score, no cut-off^a^	Composite score of Graded Symptom Scale Checklist severity and cognitive testing	No cut-off used	Clinician rated
**PCS**− **studies**										
Chong 2019 (ref. ^51^)	Longitudinal	30 (15, 15)	87	Civilian	5	Global	Total score, no cut-off^a^	Sport Concussion Assessment Tool 3	No cut-off used	Self-rated
Churchill 2019 (ref. ^52^)	Longitudinal	146 (24, 122)	50	Sports	12	Global	Total score, no cut-off^a^	Sport Concussion Assessment Tool 3	No cut-off used	Self-rated
De Souza 2020 (ref. ^53^)	Longitudinal	39 (39, 0)	26	Civilian	18	Global	Total score, no cut-off	Glasgow Outcome Scale–Extended	No cut-off used	Clinician rated
D’Souza 2020 (ref. ^48^)	Longitudinal	120 (60, 60)	43	Civilian	6	Global	Total score, no cut-off	RPQ	No cut-off used	Self-rated
McCuddy 2018 (ref. ^54^)	Longitudinal	94 (43, 51)	21	Sports	1	Mood	Total score	HAM-D	HAM-D cut-off scores	Clinician rated
**Studies that distinguished between high-symptom and low-symptom groups**										
Flowers 2021 (ref. ^59^)	Cross-sectional	64 (16 PCS+ and 16 PCS-, 32)	0	Veteran	3	Headache	Categorical cut-off score	NSI	Headache severity rating (mild (1), moderate (2), severe (3 or 4))	Self-rated
Sours 2013 (ref. ^39^)	Cross-sectional	37 (13 PCS+ and 10 PCS–, 14)	52	Civilian	1	Cognition	Diagnostic assessment + categorical cut-off score	RPQ	ICD-10 PCS diagnostic criteria + presence of cognitive symptoms on RPQ (that is, yes/no)	Clinician + self-rated

NSI, Neurobehavioral Symptom Inventory.

^a^Total score was used in neuroimaging analysis.

Across studies, there was variability in the use of clinician-rated versus self-reported measures to assess symptom burden. In the PSaC+ group, 6 out of 12 studies used clinician-rated measures, with an additional two studies incorporating both clinician- and self-rated measures, whereas 2 out of 5 PSaC– studies used clinician-rated measures (Table 1).

Several PSaC+ studies explicitly diagnosed post-concussive syndrome (PCS) (that is, the former term for PSaC) using criteria such as the tenth revision of the International Statistical Classification of Diseases and Related Health Problems (ICD-10)^15,37–39^ or *Diagnostic and Statistical Manual of Mental Disorders* fourth edition (DSM-IV)^27^, while others relied on predefined cut-off scores from their clinical outcome measures^40–45^. Three PSaC+ studies did not specify a cut-off score for symptom burden, but the scores in these studies for the PSaC patients were above suggested symptom severity thresholds in the literature (Supplementary Table 4)^26,46,47^.

PSaC– studies were primarily recovery-focused, tracking considerable improvements in primary clinical outcomes over time. At the time of neuroimaging assessment, their reported clinical outcome scores were below established cut-off thresholds for high symptom burden, including the RPQ^48–54^, Sport Concussion Assessment Tool – 3rd Edition^51,52,55,56^, Glasgow Outcome Scale–Extended^53,57^, and Hamilton Depression Rating Scale (HAM-D)^54,58^. Two studies explicitly compared high versus low symptom burden within PSaC+ groups using categorical criteria: one study categorized participants on the basis of self-reported mild versus severe headache^59^, while another distinguished between those with and without cognitive symptoms on the RPQ^39^.

In the quality assessment of the included studies, 15 of 19 were considered good quality, while 2 of 19 were deemed fair quality (1 PSaC+ and 1 PSaC− study), and 2 of 19 were rated as poor quality (both PSaC+ studies; Supplementary Tables 5 and 6). Notably, none of the included studies provided commentary on sample size justification or power description.

### Statistical analysis of coordinates derived from systematic review

A total of 177 coordinates were extracted from PSaC+ studies, compared with 67 coordinates from PSaC− studies. This difference reflects both the relatively higher number of PSaC+ studies included in the analysis and variability in the number of reported MNI coordinates across individual studies. All coordinates represented altered functional connectivity changes compared with a control group, and coordinates were pooled regardless of the resting-state fMRI methods used. In PSaC+ studies, most extracted coordinates aligned with the salience network (46, 26%), followed by default-mode network (30, 17%), visual network (28, 16%), dorsal attention network (19.5, 11%), executive-control network (17, 10%), somatomotor network (14, 8%) and limbic network (6.5, 4%). For the PSaC– studies, most extracted coordinates aligned with the default-mode network (19, 28%), followed by executive-control network (14, 21%), salience network (14, 21%), dorsal attention network (4, 6%), somatomotor network (5.5, 8%), visual network (3.5, 5%) and limbic network (4, 6%). Coordinates in the thalamus, cerebellum and midbrain could not be reliably assigned to a network for PSaC+ and PSaC− groups. We found a similar pattern of results when examining the Yeo and Schaefer atlases (80% and 83% concordance between atlases for PSaC+ and PSaC−, respectively).

Across groups, widespread multi-network changes were observed following concussion/mild TBI (mTBI; Fig. 2c). The largest difference between PSaC+ and PSaC– studies was in the salience network, with 14 of 14 (100%) of PSaC+ studies showing functional network changes compared with 4 of 7 (57%) of PSaC− studies (*P* = 0.026). Non-significant differences were also observed in the dorsal attention network (79% versus 57%, *P* = 0.24) and visual network (50% versus 29%, *P* = 0.25). The default-mode network (12 of 14, 86% versus 6 of 7, 86%, *P* = 0.48), executive-control network (12 of 14, 86% versus 6 of 7, 86%, *P* = 0.48), somatomotor network (6 of 14, 43%% versus 3 of 7, 43%, *P* = 0.36) and limbic network (4 of 14, 29% versus 3 of 7, 43%, *P* = 0.30) demonstrated similar patterns of network alteration between PSaC+ and PSaC– studies.

We next explored patterns of reported functional connectivity between the salience network and other large-scale networks in PSaC+ versus PSaC– studies. Within PSaC+ studies, 17% of salience network coordinates reported connectivity with the executive-control network, 5% with the dorsal attention network and 8% with the sensorimotor network. By contrast, 12% of salience network coordinates in PSaC– studies reported connectivity with the limbic network, compared with 4% in PSaC+ studies. The proportion of salience network coordinates linked to the default-mode network (33% versus 35%) and the visual network (10% versus 12%) was similar across both groups. These values reflect descriptive trends only, as no statistical comparisons were performed (Supplementary Table 7).

In summary, PSaC+ studies demonstrated widespread multi-network changes, with the most consistent alterations observed in the salience network, which was significantly more affected than in PSaC– studies (*P* = 0.026). While non-significant differences were noted in other networks, connectivity analyses further revealed that PSaC+ studies more frequently reported increased functional connectivity between the salience network and executive-control, dorsal attention and somatomotor networks.

### Coordinate-based meta-analysis of seed-based connectivity studies

Given the limited number of studies suitable for activation-likelihood estimation (ALE) analysis in PSaC– studies, we examined peak coordinates from PSaC+ studies using the executive-control network/dorsal attention network or default-mode network network as seed locations, as these were most represented across studies (6 studies with 32 coordinates using executive-control network/dorsal attention network seeds, and 5 studies with 23 coordinates using default-mode network seeds). ALE meta-analysis of studies using whole-brain fMRI analyses in PSaC+ with a control group (nine studies) did not reveal a statistically significant cluster of activation or deactivation when examining peak coordinates from executive-control network/dorsal attention network or default-mode network seeds. However, unthresholded ALE maps of peak coordinates from these network seeds demonstrated selective overlap with the salience network, including the bilateral insula and dorsal anterior cingulate cortex, when overlayed on the Yeo 7-network parcellation atlas (Fig. 2d).

### Computation of connectivity and brain networks

**Fig. 3:**
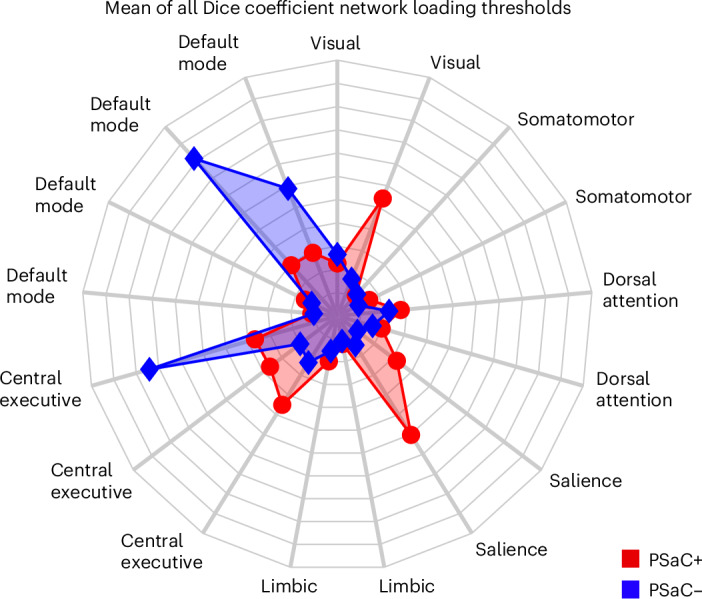
Relation between coordinates and large-scale networks in PSaC. By calculating the mean of absolute *z* scores for each Yeo network and applying Dice coefficients to the *Z* maps generated from whole-brain analyses of PSaC+ and PSaC− studies, a predominant overlap of coordinates is observed in PSaC+ studies with the salience network, in contrast to coordinates from PSaC– studies.

A network-based meta-analytic approach resulted in a robustly distributed brain network in PSaC+ that mapped primarily onto the salience network, executive-control network and visual network (Fig. 3 and Supplementary Fig. 3). By contrast, the network derived from PSaC– mapped predominantly onto the default-mode network and executive-control network.

### Deriving a network candidate for neuromodulation

**Fig. 4:**
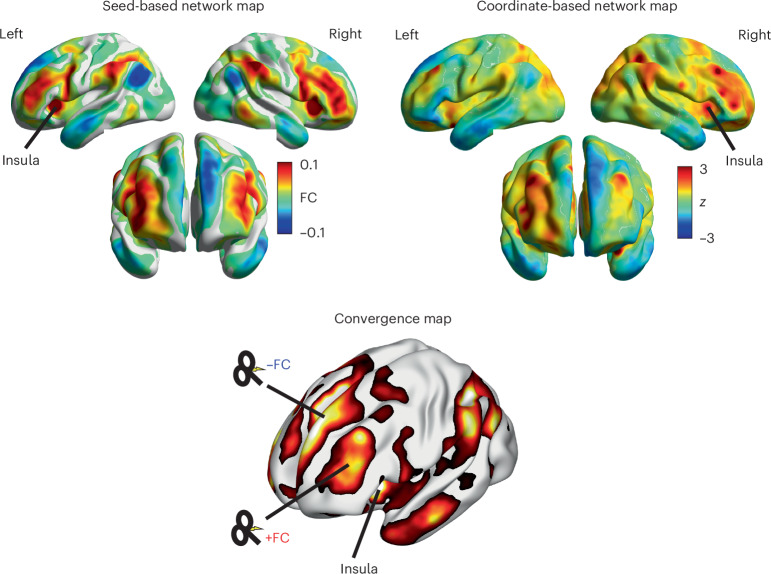
Determining candidate cortical target locations for neuromodulation. The seed-based/symptom–activation network map depicts a network map derived from existing fMRI datasets using Neurosynth. It highlights cortical areas with maximal correlation (warm colors) and anticorrelation (cool colors) with bilateral anterior insula seeds (coordinates: 34, 21, 0 for the right and –35, 20, 0 for the left). The coordinate-based network map represents a meta-analysis of coordinates derived from whole-brain connectivity PSaC+ studies. The map showcases cortical regions where network alterations are most pronounced in post-concussive syndrome (in particular, the DLPFC). The convergence map was created by voxel-wise multiplication of Neurosynth-derived seed-based and coordinate-based network meta-analytic maps, such that when the product of multiplication is positive it demonstrates convergence. Positive regions of agreement are indicated. Note that regions where there was no convergence were typically areas of weak functional connectivity or low *z* scores (that is, areas that were already not strongly implicated in either map). Two broad potential prefrontal target areas for neuromodulation are depicted. These maps can be used as seed maps to derive individual stimulation targets, as per refs. ^91,114^. DLPFC, dorsolateral prefrontal cortex; FC, functional connectivity.

Whereas the connectivity map derived from bilateral insula seeds using Neurosynth data (that is, the symptom–activation map) revealed sites of maximal correlation in the DLPFC, these locations were notably different in location from areas of maximum activation in the DLPFC generated by the network meta-analytic approach from PSaC+ studies (Fig. 4 and Supplementary Fig. 4). While the convergence map highlights potential regions of interest for neuromodulation (Fig. 4), these findings should be interpreted with caution given the broad functional role of the DLPFC and its involvement in both cognitive and affective processes. Further investigation is necessary to refine candidate targets.

## Discussion

Given the lack of convergent findings across neuroimaging literature for individuals experiencing persistent symptoms after concussion/mTBI, we aimed to (1) identify a core neural network associated with PSaC and (2) consider whether this network could be targeted at the individual level with neuromodulation. Previous work has shown that multiple systems are altered in both PSaC– and PSaC+ following concussion/mTBI. Using a converging multi-analytic approach, we identified the salience network as a core network that appears to be disproportionately altered in PSaC. Systematic review of seed-based and region-of-interest (ROI) studies also revealed increased functional connectivity between the salience network and other large-scale systems, including the executive-control network, dorsal attention network and somatomotor network, that was not observed in PSaC−. This was further corroborated by network-based meta-analysis of peak coordinates that strongly implicate salience network dysconnectivity in PSaC+, as opposed to more prominent executive-control network and default-mode network changes seen in PSaC– studies (Fig. 3 and Supplementary Fig. 3). This underscores the importance of the salience network and network-level considerations in understanding the pathophysiology of PSaC. To investigate personalized treatment approaches for PSaC, we used the salience network regions derived from the PSaC symptom–activation analysis to locate connected cortical regions amenable to non-invasive brain stimulation (Fig. 4). A DLPFC region displaying maximal connectivity to these ROIs was robustly identified, aiding in the determination of an optimal candidate target for delivering personalized brain stimulation treatment in PSaC trials.

The salience network^60,61^, also known as the cingulo-opercular network^62^, ventral attention network^63^ or midcingulo-insular network^64^, has been described as a ‘neural crossroads’ due to its extensive connectivity and influence on other networks^65^. Functionally, it plays a central role in perceiving and responding to internal signals and orchestrating shifts in attention when the importance of signals changes^66,67^. Core network regions include the anterior insula and anterior midcingulate cortex, with additional cortical (for example, supramarginal gyrus, temporoparietal junction and a component of the middle frontal gyrus) and subcortical structures (for example, amygdala, hypothalamus, putamen, caudate, nucleus accumbens and periaqueductal gray) contributing to its function^61,67^. Dysfunction of this network has emerged as a consistent transdiagnostic feature across neuropsychiatric disorders^65,68,69^, and efficacy of non-invasive brain stimulation methods, such as rTMS in treatment-resistant depression, has recently been associated with connectivity changes in the salience network^70,71^. Abnormalities in the salience network have been linked to transdiagnostic neuropsychological factors, such as somatosensory amplification^72,73^, hyperarousal-associated insomnia^74^, fear-avoidance behavior^75^ and mismatch between self-appraisals of objective cognitive performance and symptom reporting^76^, which have been identified as relevant predisposing/perpetuating factors in PSaC^7–9,18,77^. Furthermore, it has been postulated that initial symptoms induced by concussion (for example, dizziness, headache, fatigue, mood dysregulation and cognitive difficulties), which are typically transient, may get amplified and sustained in the subset experiencing PSaC due to excess attention on physical symptoms^1,9^. The neurophysiological role of the salience network is well positioned to be a critical mediator of such a process^66,76,78^; however, whether this may be due to pre-existing network vulnerabilities, network changes induced by the concussion or a combination of both, remains incompletely understood.

The first part of this study involved spatially combining functional PSaC symptom maps that approximated to RPQ clinical symptoms. The union of these maps converged within the salience network (Fig. 2b). PSaC symptom maps were derived from a symptom-based approach rather than an etiological-based approach, meaning they were not based exclusively on TBI populations. However, the convergence of multiple different functions on the salience network, whether related to TBI specifically or not, implicates a multimodal sensory processing and integrative nexus that may be particularly vulnerable to post-injury remodeling^79^. To improve specificity, we conducted a coordinate-based meta-analysis using Neurosynth Compose, filtering for studies that included exclusively participants with concussion. The resulting binarized activation map was intersected with the union of the 11 RPQ symptom–term activation maps, ensuring that only co-localizing regions were retained. This confirmed that the identified areas of activation remained co-localized to the bilateral insula and salience network, reinforcing the relevance of this network to PSaC.

PSaC+ studies from our systematic review, where salience network dysfunction was identified, involved a range of symptoms, such as pain/headache^37,41,59^, vestibular and visual symptoms^42,47^, cognition^39,44^ and mood symptoms^43^, with the remainder involving global symptom burden measured by PSaC symptom questionnaires^26,27,38,40,45,46^. Although not a focus of our study, early post-injury changes in salience network structures may potentially differentiate those who develop chronic symptoms from those who recover^23,74,76,78,80^. Recent research by Woodrow et al.^14^ implicated early functional alterations in the right ventral anterior and ventrolateral dorsal thalamic nuclei as distinguishing factors in PSaC, despite the absence of structural damage on CT imaging. Whereas the anterior thalamus and dorsomedial thalamus have been previously associated with the salience network, Kawabata et al.^81^ found that the ventral anterior and ventral lateral thalamus exhibited the strongest connections with salience/cingulo-opercular networks. In addition, changes in salience network connectivity over time appear to be associated with symptom recovery following mTBI^51,82^. Given the diverse symptomatology associated with salience network dysfunction in PSaC, it prompts further investigation into the network’s role in symptom generation and persistence.

On the basis of included studies employing a seed-based connectivity approach, PSaC+ studies were found to have selectively increased functional connectivity between bilateral DLPFC and multiple salience network nodes compared with PSaC–, including the right insula, right amygdala/putamen, supplementary motor cortex and left supramarginal gyrus^26,27,38,39^. These regions have been linked to neuropsychological factors commonly observed in PSaC, including altered interoceptive awareness^83^, heightened sensory sensitivity^21^ and diminished cognitive control, respectively^84^. By contrast, reduced functional connectivity was found between the right DLPFC and left midbrain (salience network)^44^, as well as right supramarginal gyrus (salience network) and bilateral posterior parietal cortex (executive-control network), potentially reflecting diminished top-down regulation of sensory and attentional systems^27,85^. For salience network–dorsal attention network connectivity, increased connectivity was observed between the right supramarginal gyrus and the right superior parietal lobule (areas involved in working memory and attentional control) and the multisensory processing area of the temporoparietal junction^40,47^. In addition to changes in the salience network being discovered in significantly more PSaC+ studies than PSaC– studies (*P* = 0.026), these findings highlight a unique connectivity profile that further characterizes individuals with PSaC. These patterns include features linked to symptom preoccupation and hypervigilance, which are core to somatization, a factor strongly associated with salience network dysfunction and risk of developing PSaC^9,72^.

PSaC+ and PSaC− studies were not differentiated by the degree of salience network–default-mode network connectivity (Supplementary Table 7), but this network pairing represented the majority of extracted coordinates from seed-based and ROI–ROI functional connectivity studies (33%) and was the most commonly investigated network a priori across included studies (Supplementary Table 2). Among the PSaC+ studies, reduced anticorrelation between the default-mode network and the salience network (including bilateral insula, left premotor cortex and bilateral supramarginal gyrus) was identified^39,40^. Interactions between salience network–default-mode network on inefficient cognitive control in patients with cognitive impairment following TBI have been previously reported by Bonnelle et al.^22^ However, default-mode network changes in PSaC have not been consistently found^23,27,42,44^. More recently, Trapp et al.^86^ conducted a large-scale investigation examining the relationship between penetrative TBI in veterans and persistent depressive symptoms, revealing that damaged regions within the salience network, such as the bilateral anterior insula and dorsolateral prefrontal cortex, were identified as ‘risk’ regions associated with higher depression severity. Conversely, structurally damaged regions within the default-mode network, including the right orbitofrontal cortex and medial prefrontal cortex, were identified as ‘resilience’ regions associated with lower depression severity. It is interesting to note that our network meta-analysis revealed greater default-mode network, but not salience network, activation in PSaC– studies, whereas PSaC+ studies revealed the opposite.

Regarding the coordinate-based ALE analysis, the lack of statistically significant spatial clustering is not in line with previous findings^87,88^. Importantly, these studies included task-based fMRI, whereas our study included only resting-state fMRI results. In addition, our meta-analysis of resting-state fMRI whole-brain studies was probably underpowered to detect significant effects. The number of studies meeting criteria for ALE (*n* = 7) was below the recommended range of experiments (*n* = 17–20) to yield adequate statistical power in simulations^89^. Lack of statistically significant spatial clustering could also have been due to heterogeneity between studies in terms of demographics (sex, age, comorbidities, veteran versus civilian versus athlete), clinical characteristics (time from injury to scan, degree of symptomatology) and fMRI procedures (statistical thresholding/correction, ROI selection, resting-state method use (seed-based analysis versus independent component analysis versus ROI–ROI); (Table 1 and Supplementary Table 2)). However, unthresholded ALE maps revealed a pattern of disruption with notable localization to the salience network. Thus, despite these factors, multiple lines of analysis in our study have convergent findings in the salience network.

Other notable network changes outside the salience network in PSaC+ studies include the somatomotor and visual networks, both of which were found to have increased connectivity with all networks and distinguished PSaC+ and PSaC− studies (Fig. 2c). Given the presence of visual, vestibular and pain symptoms commonly reported in PSaC, it is not surprising to see hyperconnectivity and hyperactivation among these networks^40,42,90^. The visual network was also found to have considerable activation across binarized *z*-map thresholds in the network-based meta-analytic approach of PSaC+ studies, which was not found across PSaC– studies (Fig. 3).

The DLPFC has been most commonly targeted in PSaC, although targeting has relied on scalp-based heuristics^28^. Our study may help inform DLPFC targets for PSaC. To this end, we derived network maps using two separate approaches: we derived (1) a whole-brain functional connectivity map from the anterior insula cluster identified in the symptom-based network analysis using Neurosynth data and (2) a coordinate-based network utilizing all previously reported activation/deactivation coordinates from resting-state functional connectivity studies examining those with PSaC+ compared with PSaC– following concussion/mTBI. Both approaches converged to implicate the salience network (Figs. 2 and 3 and Supplementary Fig. 3). Our Neurosynth seed-to-voxel connectivity analysis using the bilateral anterior insula as a seed region demonstrated regions of both positive and negative functional connectivity across the DLPFC and anticorrelated with regions of the default-mode network, such as the medial prefrontal cortex and angular gyrus (Fig. 4). Notably, while the DLPFC is a common stimulation target in depression, it is a heterogeneous region with functionally distinct subregions^91^. The DLPFC target identified in our PSaC analysis localized more medially compared with the typical lateral DLPFC region targeted using Beam F3 in depression protocols (Fig. 4)^92^. This distinction may reflect the broader symptom profile of PSaC, which extends beyond mood symptoms to include cognitive, sensory and somatic complaints. The PSaC-linked circuit identified here therefore maps onto a wider constellation of symptoms than dysphoria alone.

The seed-based connectivity map generated from the bilateral anterior insula/salience network cluster identified from the Neurosynth analysis demonstrated close agreement with the coordinate-based network map, as indicated in the convergence map (Fig. 4). Regions where there was no convergence were typically areas of weak functional connectivity in the Neurosynth map and low *z* scores in the coordinate-based network map (that is, areas that were already not strongly implicated in either analysis). These analyses indicated two broad regions of maximal convergence in the prefrontal cortex, implicating potential brain stimulation targets of positive and negative functional connectivity. The most clinically effective combination of target region and TMS frequency will require further investigation. Cortical sites of both negative and positive connectivity to downstream regions have been implicated in clinical response^93,94^. A further consideration is that it remains uncertain whether these distinct DLPFC regions represent a causal or compensatory network in PSaC, and whether stimulating these areas would prove beneficial or potentially deleterious to the recovery process.

Finally, a major goal has been to personalize care in PSaC, a challenge due to its heterogeneity^4,5^. The identification of a core network of dysfunction accessible by personalized rTMS offers a therapy that can potentially be used across PSaC presentations. The application of individualized resting-state network mapping has demonstrated improved accuracy of stimulation protocols for TBI-related depression, potentially enhancing TMS treatment efficacy^15,35^. However, robust clinical data utilizing this approach on outcomes in PSaC/TBI-related depression are lacking. Moreover, it is worth noting that TBI-associated depression has been characterized primarily by increased connectivity between the default-mode network and dorsal attention network, as well as enhanced connectivity between the dorsal attention network and the subgenual anterior cingulate cortex^95^. The dorsal attention network was found to be disrupted in almost 80% of included PSaC+ studies (versus 57% in the PSaC– group), although this difference was not statistically significant. Our network-based meta-analysis of coordinates from studies using whole-brain connectivity did not find spatial overlap with the dorsal attention network in either PSaC+ or PSaC– studies. This discrepancy may speak to the heterogeneity and complexity in considering the broader PSaC category, as opposed to more selective phenotyping of post-TBI depression. Of note, the study by Siddiqi et al.^95^ included samples of more severe TBI than concussion, which may also account for the discrepancy. Overall, given the lack of effective treatments and early stage of neuromodulation approaches for TBI, new network targets that may be aligned with the underlying pathophysiology of PSaC are needed to guide future treatment trials.

## Limitations

There are many limitations that need to be highlighted. First, the RPQ symptom–activation maps derived from Neurosynth meta-analytic data involved task-based fMRI studies with non-TBI participants, and the findings could be confounded by comorbidities unrelated to PSaC. Meta-analytic data were not available for several RPQ symptom domains (nausea/ vomiting, dizziness/imbalance, blurred/double vision and processing speed). It is possible that the union of multiple task-based fMRI studies, regardless of condition, could involve salience network activation as a common feature^66^. However, incorporating a concussion-specific meta-analytic activation map derived using Neurosynth Compose and filtered to include only concussion studies yielded stable results when combined with the RPQ symptom–activation map, reinforcing the association between PSaC symptoms and salience network topology. Disruption of a pivotal hub that coordinates diverse functions across domains emphasizes the role of the salience network in understanding the various symptoms in PSaC.

Second, our network mapping approach was limited to studies that reported coordinates. Within the studies included in our systematic review, there was substantial heterogeneity in the categorization, diagnosis and neuroimaging evaluation of PSaC. For example, there were proportionally more longitudinal prospective studies in the PSaC– group of studies, which may have a lower likelihood of capturing high-symptom PSaC over time as compared with cross-sectional studies focusing on high-symptom PSaC, which would introduce bias in our results. Regarding control groups, PSaC+ studies primarily used healthy controls, whereas PSaC– studies predominantly used a prospective design with comparisons between acute injury and follow-up assessments, often without a healthy/non-TBI control group. Although our intention was to include studies that classified patients as recovered or having low symptom burden, it is possible that some individuals in these groups were still recovering or experiencing residual PSaC symptoms. This variability in symptom severity and recovery stage among patients may have introduced confounding factors that influenced resting-state functional connectivity results.

In addition, there were relatively fewer low-symptom/recovery studies, and only three that used whole-brain analysis, which precluded ALE meta-analysis for PSaC−, thus limiting comparisons with PSaC+ studies. Notably, half of the MNI coordinates that mapped to the salience network across PSaC– studies were derived from a single study^54^, whereas for the PSaC+ studies the highest contribution from a single study was 15% (ref. ^26^). However, this observation further highlights the convergent evidence across studies linking the salience network to PSaC+. The results of the network-based meta-analysis are limited by the relatively low number of studies using non-seed-based whole-brain connectivity approaches. Among the PSaC+ studies, 60 coordinates from 5 studies were used, whereas for PSaC– studies, only 9 coordinates from 3 studies could be used. This imbalance may reflect a combination of factors, including our stringent inclusion criteria (for example, resting-state fMRI data collected at least 1 month post-injury, extractable activation coordinates), the availability of usable data for analysis, or broader trends in the neuroimaging literature that emphasize studies of persistent symptoms^20^.

While our study included individuals with chronic persisting symptoms irrespective of mechanism of injury, we acknowledge that blast-related mTBI may differ from sports-related concussion in terms of symptomatology and psychiatric risk factors^96,97^. However, the proportion of coordinates from veteran populations was relatively balanced between PSaC+ and PSaC– studies, and the included studies did not focus exclusively on blast-related injuries. Thus, while these differences should be considered in the interpretation of findings, we do not believe they bias the results.

Last, regarding potential therapeutic target sites, the incongruity between the seed-based connectivity map and network meta-analysis raises critical questions about the functional importance of these discrepant DLPFC areas in the context of PSaC. Specifically, it remains unclear whether these discrepant areas within the DLPFC represent a causal or compensatory network in PSaC. In addition, the potential consequences of stimulating these areas on the clinical recovery of PSaC patients remain to be explored. Future studies are recommended to include a retrospective validation or lesion-based network approach.

## Conclusions

Our study sought to explore the core neural underpinnings of persisting symptoms after concussion (PSaC) to identify a canonical network potentially suitable for targeted neuromodulation therapies. Our findings underscore the salience network as a neural hub that is disproportionately affected in PSaC across multiple analytic approaches, serving as an anchor for core PSaC symptoms. Personalized network mapping using data from the Human Connectome Project identified an area of the DLPFC maximally co-activated/correlated with the salience network as a candidate target for tailored brain stimulation interventions. It is essential to interpret these results cautiously due to limitations in symptom–activation map generation, availability of data in published literature, heterogeneity across included studies and lack of retrospective validation. Nevertheless, the consistent emergence of the salience network in PSaC+ studies underscores its pivotal role in chronic symptoms following concussion/mTBI, offering promise for the development of targeted therapeutic interventions in PSaC within the evolving field of brain stimulation.

## Methods

### PSaC symptom-based network mapping

To identify a functional network that is clinically pertinent to PSaC, we used the Neurosynth database (http://www.neurosynth.org) to extract meta-analytic activation maps from search terms approximated to symptoms on the 16-item RPQ. The RPQ is a diagnostic tool commonly used to diagnose PSaC and includes somatic, affective, cognitive and behavioral components, with higher scores reflecting higher symptom burden^50,98^. The homologous RPQ search terms were identified in the Neurosynth database (Fig. 2a) and included ‘anxiety’, ‘depressive disorders’, ‘visual stimuli’, ‘auditory stimuli’, ‘impulsivity’, ‘angry’, ‘memory’, ‘concentration’, ‘pain’, ‘sleep’ and ‘arousal’. The Neurosynth database contains 14,371 whole-brain human fMRI studies and automatically computes Pearson correlation coefficients between submitted whole-brain maps and 1,334 terms used in contributed publications. Neurosynth computes these activation maps by performing meta-analyses across thousands of whole-brain fMRI studies using a uniformity test (a one-way analysis of variance) to identify voxels that are consistently reported as active in studies using a given term. These maps represent the spatial distribution of activation consistently associated with each term and are publicly available for download. Each activation map was binarized in FSLeyes (Functional Magnetic Resonance Imaging of the Brain (FMRIB) Software Library (FSL),version 1.6.1, 2023)^99^ by assigning a value of 1 to all voxels with nonzero *z* scores, and 0 otherwise, to reflect the spatial extent of consistent activation associated with each term. The union of these binarized maps was then used to compute common regions of functional activation across approximated RPQ symptom–term domains.

These neuroimaging studies did not include primarily patients with mTBI (Supplementary Table 8). To further refine the symptom-based network map with data from a more clinically specific population, we conducted a targeted meta-analysis using Neurosynth Compose, a new tool that allows for study-level filtering. We selected 37 studies explicitly involving participants with concussion, yielding a total of 57 contrasts and 847 reported MNI coordinates. We conducted a coordinate-based meta-analysis using the MKDAChi^2^ algorithm with the following parameters: kernel radius of 10 mm, kernel value of 1 and prior probability of 0.5. Multiple comparisons correction was performed using a false discovery rate corrector (*α* = 0.05, independent method). The resulting *z*-score activation map was binarized using FSLeyes and overlaid with the union of all RPQ symptom–activation maps. The full meta-analysis output is publicly available^100^.

### Systematic review of PSaC resting-state-based network mapping

To investigate whether our RPQ symptom map co-localized to PSaC network dysfunction reported in the literature, we conducted a systematic review of PubMed and MEDLINE databases for all studies published between January 2000 and September 2023 that examined resting-state fMRI in mTBI or concussion. We also searched reference lists of reviewed papers to identify other relevant papers. Studies were categorized into PSaC+ or PSaC− focused. To differentiate between high and low symptom burden in PSaC studies, we classified studies on the basis of their primary clinical outcome measures at the time of neuroimaging. Studies that explicitly diagnosed PCS using standardized criteria (for example, ICD-10 or DSM-IV) and/or applied predefined cut-off scores for symptom severity (for example, RPQ > 30, HAM-D > 8, CAPS > 2) or categorical distinctions (for example, absent/mild versus severe on a rating scale) were classified accordingly. When no clear cut-off was provided, we determined symptom burden grouping by assessing whether reported scores on primary clinical outcomes exceeded (PSaC+ studies) or fell below (PSaC– studies) recognized thresholds associated with greater symptom severity and poor recovery. These classifications were systematically applied across all included studies (Supplementary Table 4). The rationale for diagnosing PSaC on the basis of >1 month of persistent symptoms was supported by several studies^3,27^.

Articles were reviewed for inclusion by two independent researchers (A.M. and I.J.S.), and discrepancies were resolved by S.M.N. This systematic review followed PRISMA guidelines (see PRISMA checklist in Supplementary Methods for further detail). We included articles that (1) contained original, peer-reviewed research, (2) involved adult participants and (3) collected resting-state fMRI data at least 1 month following mTBI. Data extraction, including clinical, methodological and demographic variables, was completed by two investigators (A.M. and C.F.D.L.). Quality assessment of the included studies was conducted by two investigators (A.M. and C.F.D.L.) using the quality assessment tools for Observational Cohort and Cross-Sectional Studies and Controlled Intervention Studies^101^.

### Statistical analysis of coordinates derived from systematic review

To assess network differences associated with persistent symptoms, we compared the distribution of activation coordinates from PSaC+ versus PSaC– studies by mapping their reported peak coordinates onto canonical large-scale brain networks using FSLeyes (version 1.6.1, 2023). In addition, two studies directly compared high versus low symptom burden PSaC groups and were categorized accordingly (Supplementary Table 2).

For each study, we extracted peak activation coordinates of regions showing statistically significant between-group differences with controls. Talairach coordinates were converted to MNI space using GingerALE’s Talairach-to-MNI conversion tool (http://brainmap.org/ale/)^102,103^. To determine affected networks, all peak MNI coordinates derived from the systematic review were overlayed on the Yeo 2011 7-network atlas and Yeo/Schaefer 2018 17-network atlas using Schaefer 400- and 1,000-parcel resolutions in FSLeyes^63,104^. Each coordinate was assigned a value of 1 for a single-network assignment or 0.5 if it spatially overlapped two adjacent networks. This approach allowed for equal representation of coordinates near network boundaries. While activations and deactivations were documented separately for descriptive purposes, the same mapping and scoring procedure was applied to both. The full list of MNI coordinates extracted during systematic review is publicly^105^.

This mapping allowed us to estimate the distribution of activation and deactivation peaks across large-scale brain networks. To enable group-level comparisons, we then binarized network involvement at the study level: if a given network contained at least one assigned coordinate (1 or 0.5), it was coded as ‘1’ (altered) for that study; otherwise, it was coded as ‘0’ (unaffected). This allowed us to perform a Fisher’s exact test comparing the frequency of network involvement between PSaC+ and PSaC– studies. A similar methodology was employed in ref. ^106^. Analyses were performed in SPSS (version 29, IBM Corp.)^107^.

### Coordinate-based meta-analysis of network stability

To evaluate whether there was statistically significant spatial clustering of coordinates across PSaC+ studies, we performed a coordinate-based meta-analysis using ALE. This method identifies regions where reported activation peaks from different studies converge spatially more than would be expected by chance, highlighting localized effects. While this identifies potential regional spatial convergence, it does not directly assess the broader network-level connectivity patterns associated with these coordinates. The ALE method was implemented via GingerALE (version 3.0.2; http://brainmap.org/ale/). This method has been previously described^108^. All analyses were performed in MNI space, accounting for multiple comparisons with a cluster-forming threshold of *P* < 0.001 (uncorrected) and a cluster-level family-wise error rate of 5% (ref. ^109^). Given the limited number of PSaC– studies, this analysis was performed on PSaC+ studies that used a seed-based, whole-brain voxel approach with default-mode network or executive-control network as the seed network. The goal was to determine whether any significant activation clusters emerged and, if so, to identify the associated network. Studies using ROI-to-ROI connectivity (that is, non-whole-brain analyses) were excluded from ALE analysis.

### Network-based meta-analysis of network stability

Another approach to determining which network the coordinates derived from the systematic review predominantly mapped onto involved a network-based meta-analytic approach. Unlike ALE, which relies on the spatial convergence of activation clusters, this method assesses which functional networks are preferentially involved by examining the distribution of coordinates across the whole brain and their connectivity patterns within a large normative dataset.

As an overview, coordinates were derived as described in the preceding, but only experiments utilizing whole-brain group analyses were included; those utilizing ROI seed-based analyses were excluded (see Supplementary Table 2 for details on which studies employed whole-brain analysis and where coordinates were derived from). Next, whole-brain functional connectivity was computed for each coordinate using resting-state fMRI images from 100 healthy adults participating in the HCP^110,111^. These networks were summed and divided by the total number of constituent coordinates to generate a mean functional connectivity map. PSaC+ and PSaC– functional connectivity maps were then subjected to statistical testing against a null distribution and converted to *z*-scored brain maps (Supplementary Fig. 3)^112^.

We analyzed data from the first two fMRI sessions in the HCP database, acquired consecutively on the first day of scanning. Each of the two data acquisition sessions comprised 14 min, 33 s runs (right-to-left and left-to-right phase encodings, 1,200 volumes each), with eyes open and relaxed fixation on a projected bright crosshairs on a dark background (and presented in a darkened room). These two 14 min, 33 s runs per day were temporally concatenated (following preprocessing, described next) to result in 29 min of data and 2,400 datapoints per concatenated scan. To minimize temporal discontinuity, the mean was removed from each time series before concatenation. For additional details regarding concatenation and the HCP functional preprocessing pipeline, see Supplementary Methods section Network-based meta-analysis.

Spherical seeds (4 mm radius) were centered at each reported coordinate. Whole-brain functional connectivity maps were then computed by correlating the mean time series from this sphere with time series for every voxel composing a gray-matter mask (FSL MNI 152 2 mm brain template). The functional connectivity maps from all coordinates in a given condition (that is, PSaC+ and PSaC–) were summed voxel-wise, and the summand was divided by the total number of constituent coordinates to generate a condition-specific functional connectivity map. This procedure was repeated for 100 individuals, and the resulting functional connectivity maps were averaged to generate a single normative functional connectivity map for each condition.

Next, the mean functional connectivity map for each condition (PSaC+ and PSaC–) was subjected to statistical testing. This procedure mimicked that described in the preceding except that functional connectivity maps were derived from randomly generated (rather than real) coordinates uniformly distributed throughout the gray-matter volume. The same sample of 100 individuals from the HCP was used to compute functional connectivity maps. For a given condition, the number of contributing coordinates (***n*** coordinates) was also held identical across observed (real) and randomly generated (null) functional connectivity maps. This procedure was repeated 1,000 times per condition to generate 1,000 group-average functional connectivity maps, each derived from ***n*** coordinates. We used this empirical null distribution to test the alternative hypothesis that the observed coordinates were constrained to specific functional networks. To this end, a *z*-score map was computed by subtracting the mean of the 1,000 null samples from the observed functional connectivity map and dividing the result by the standard deviation across the 1,000 null samples. This was performed independently for each gray-matter voxel and condition, yielding a z-score map for each condition. These *z*-scored condition-specific brain network maps are referred to as ‘brain maps’. In simpler terms, these brain maps provide a representation of the connectivity of dysfunctional brain regions in individuals with TBI presenting with (PSaC+) or without (PSaC−) PSac, and every voxel value corresponds to the level of deviation from what would be expected by chance (as defined by the null distribution).

To understand the etiology of the brain maps derived, we next computed spatial alignment with canonical (Yeo-7) functional networks (Supplementary Fig. 3). The Dice correlation coefficient was utilized for this purpose as it allows computation of spatial similarity with a binarized network (that is, with Yeo networks). Given that the arbitrarily selected threshold necessary to binarize PSaC+ and PSaC– networks might influence spatial correlations, this computation was performed with absolute *z* maps thresholded at 10, 15, 20, 25 and 30% increments (Supplementary Fig. 3). The mean spatial correlation was computed across these thresholds.

### Deriving neuromodulation targets for PSaC

Last, to determine whether a cortical target for neuromodulation could be identified within the network implicated in PSaC, we examined the functional connectivity of the key regions identified in the previous analyses. This approach aimed to establish a site within the affected network that could serve as a potential intervention target on the basis of its connectivity profile and convergence across multiple mapping methods.

A functional connectivity map was derived from the two bilateral anterior insula clusters identified in the preceding in the Neurosynth symptom-mapping step using minimally preprocessed data from the HCP (*N* = 1,000 concatenated 28-min resting-state fMRI scans) and previously detailed methods^91,111,113^. This involved (1) deriving the mean time series across both clusters for each participant; (2) computing a whole-brain functional connectivity map for each participant; and (3) generating a group-average functional connectivity map. We computed the convergence of this map with the coordinate-based network brain map generated from PSaC+ coordinates derived above from our systematic review (Supplementary Fig. 4). The convergence map was created by voxel-wise multiplication of Neurosynth-derived seed-based and coordinate-based network meta-analytic maps, such that when the product of multiplication is positive it demonstrates convergence.

### Reporting summary

Further information on research design is available in the Nature Portfolio Reporting Summary linked to this article.

## Supplementary information

Supplementary Information Supplementary Figs. 1–4, Tables 1–8 and Methods (Network meta-analysis).

Reporting Summary

## Data Availability

The full meta-analytic output from the Neurosynth Compose analysis is publicly available at https://identifiers.org/neurovault.collection:20829 (ref. ^100^) The dataset supporting the findings of this study (that is, the full list of MNI coordinates extracted during the systematic review) is publicly available at https://osf.io/dpzmy/?view_only=4e9a8c80d37c464f98ba8cc7521a43d7 (ref. ^105^).
