# Supplementary material for: The network-based underpinnings of persisting symptoms after concussion: a multimodal neuroimaging meta-analysis
Source: Nat Ment Health. 2025 Sep 23;3(10):1276–90. doi: 10.1038/s44220-025-00503-6 (PMC12504115; doi:10.1038/s44220-025-00503-6)
Supplement: Supplementary file 1 — Supplementary Figs. 1–4, Tables 1–8 and Methods (Network meta-analysis). [file 44220_2025_503_MOESM1_ESM.pdf]

nature mental health

Supplementary information

https://doi.org/10.1038/s44220-025-00503-6

The network-based underpinnings of persisting symptoms after concussion: a multimodal neuroimaging meta-analysis

In the format provided by the authors and unedited

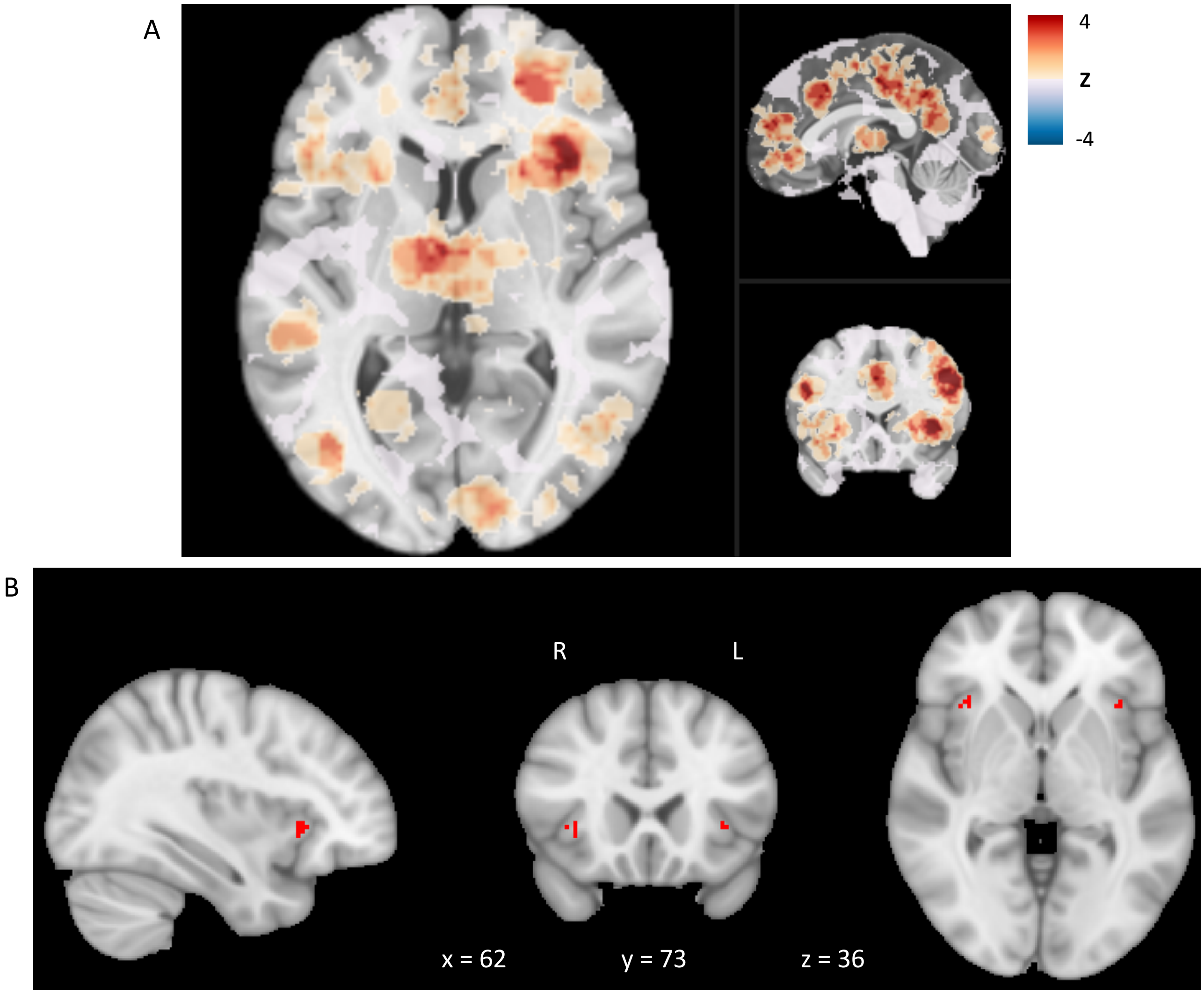

Supplementary Figure 1: Meta-analytic activation maps from Neurosynth Compose and symptom-overlap analysis. (A) Spatial activation map (z-scores) derived from a Neurosynth Compose meta-analysis of 37 concussion-specific studies, visualized in horizontal, sagittal, and coronal planes. (B) Binary overlap map created in FSLeyes showing the intersection of the binarized concussion-specific activation map from panel A and the union of 11 RPQ symptom-term activation maps. Convergent regions localize to the right and left anterior insula, consistent with salience network topology.

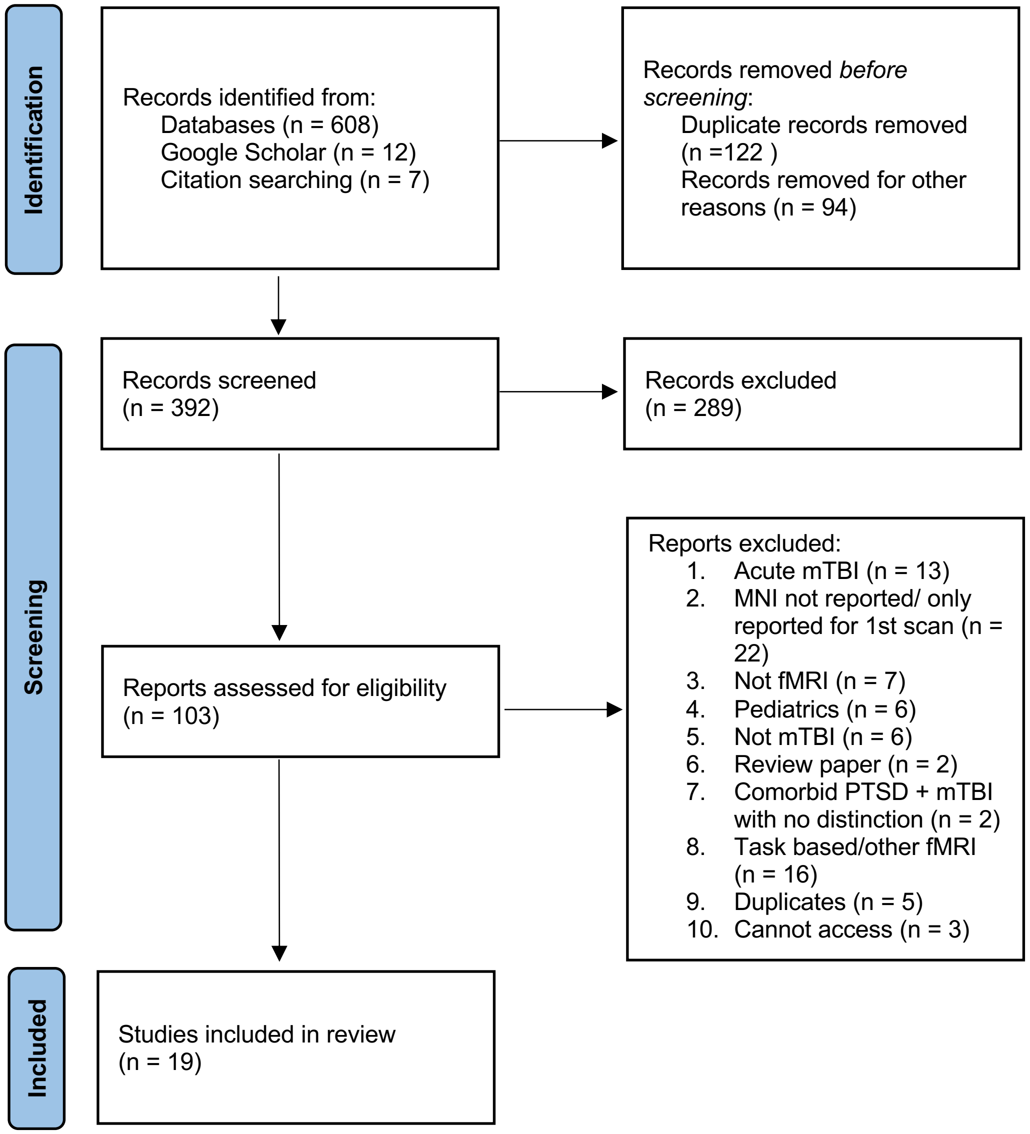

Supplementary Figure 2: PRISMA flow diagram for the selection of resting state functional magnetic resonance imaging studies of post-concussive syndrome or chronic mild traumatic brain injury.

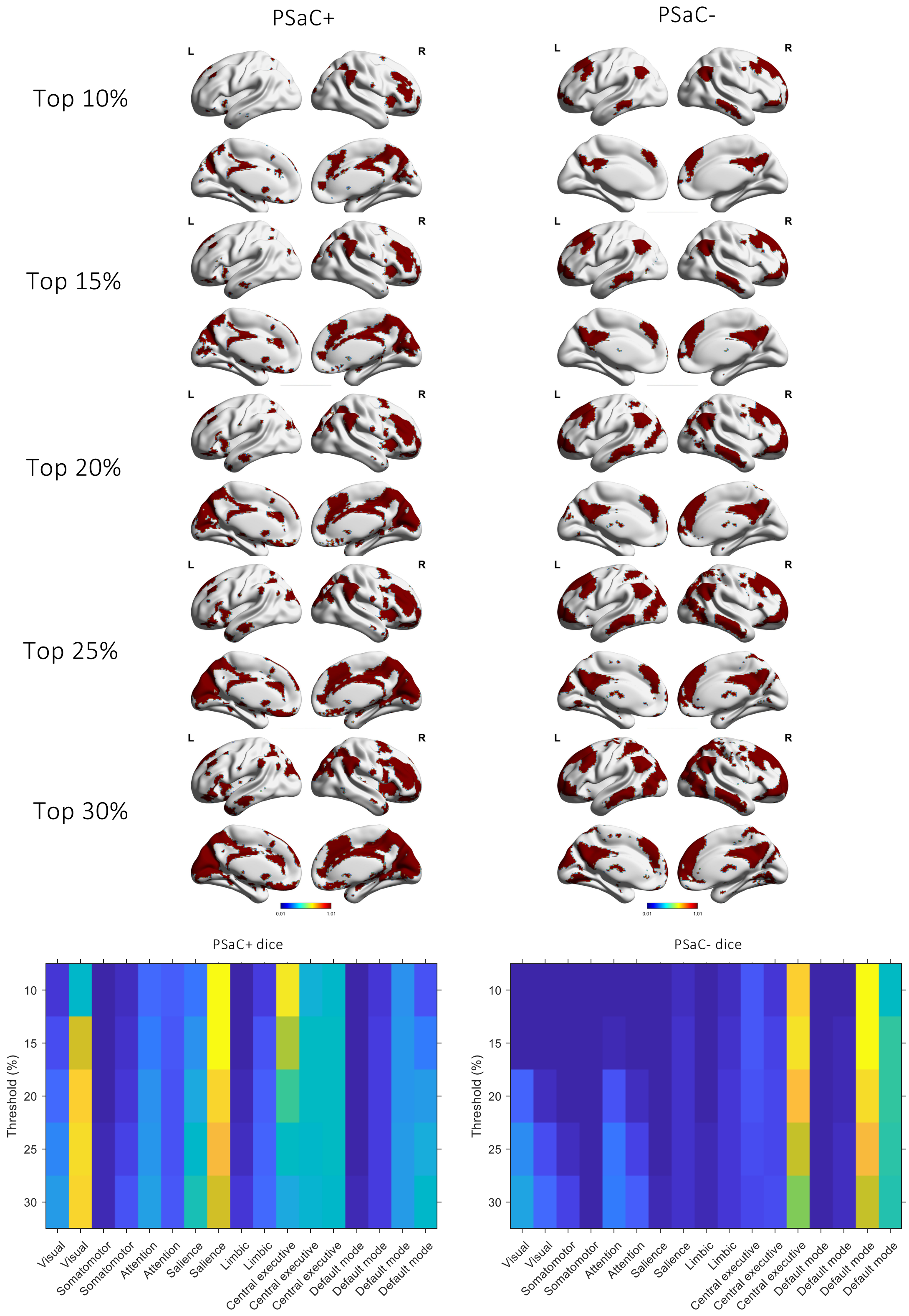

Supplementary Figure 3: Binarized Z-maps generated from coordinates extracted from whole brain analyses PSaC+ and PSaC- studies. Z-maps were thresholded at 10, 15, 20, 25 and 30% increments and binarized to retain the top percentage of voxels. Network loading through the application of Dice coefficients on Z-maps generated from coordinates extracted from whole brain analyses PSaC+ and

PSaC- studies. Spatial alignment with canonical (Yeo-7) functional networks was computed. Given that the arbitrarily selected threshold necessary to binarize PSaC+ and PSaC- networks might influence spatial correlations, this computation was performed with absolute z-maps thresholded at 10, 15, 20, 25 and 30% increments. PSaC+ study coordinates primarily mapped onto salience network, central executive/executive control network, and visual network. In comparison, the PSaC- derived network mapped predominantly onto the default mode and central executive/executive control networks.

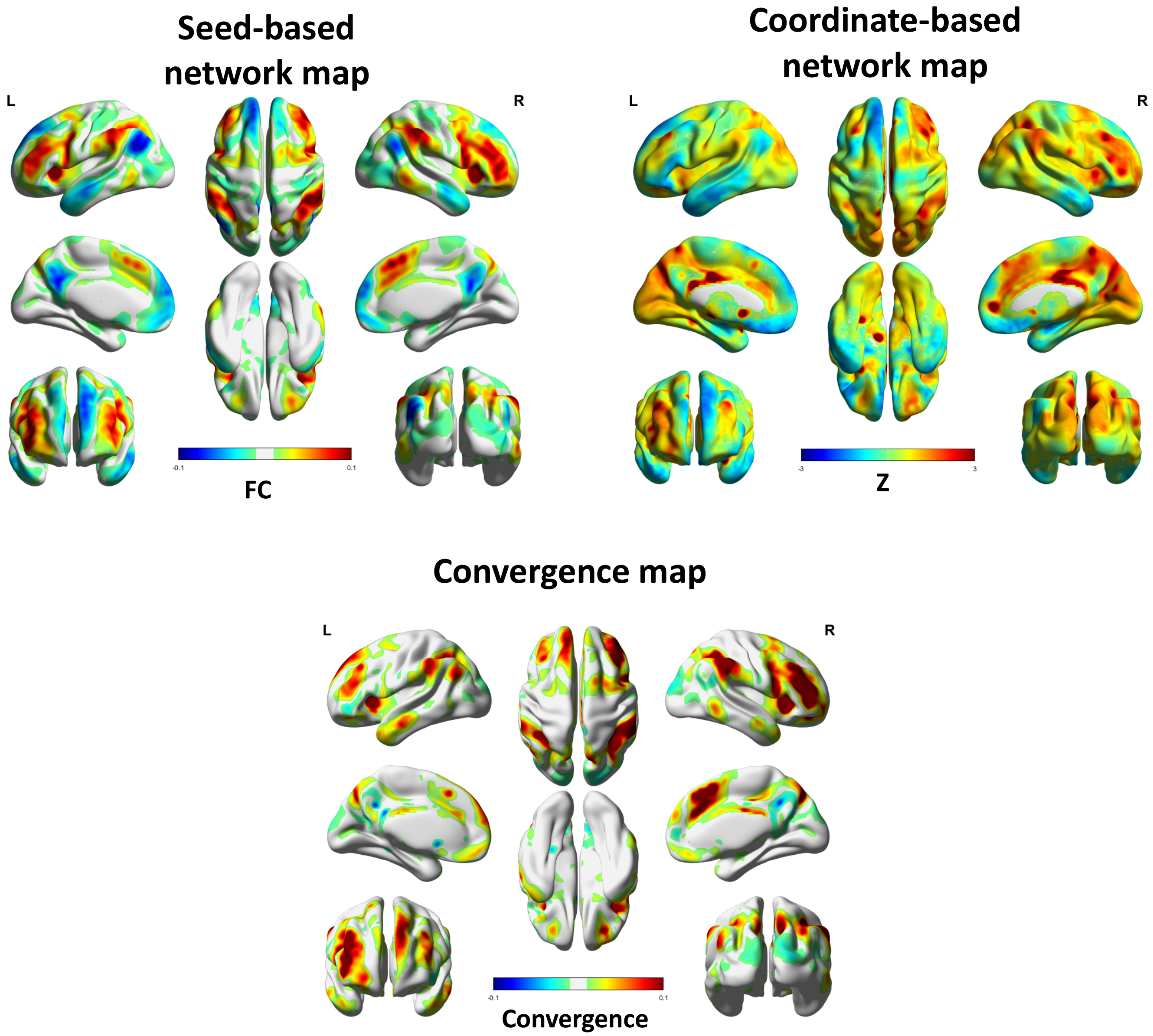

Supplementary Figure 4: Cortical Network Maps in PCS. Seed-Based/Symptom-Activation Network Map: depicts intensity of functional connectivity derived from fMRI datasets using Neurosynth. Cortical areas are shown with maximal correlation (warm colors) and anticorrelation (cool colors) with bilateral anterior insula seeds (coordinates: 34, 21, 0 for the right and -35, 20, 0 for the left). **Coordinate-Based Network Map:** represents a meta-analysis of Z scores from whole brain connectivity studies in PCS+ participants, showcasing regions with pronounced network alterations, particularly in the dorsolateral prefrontal cortex. **Convergence Map:** created by multiplying the seed-based and coordinate-based maps, indicates the degree of convergence. Positive values (warm colours) highlight regions of agreement, while negative values (cool colours) denote areas of no agreement between the maps.

Supplemental Table 1: Pertinent descriptive results from included studies.

Study	Pertinent activation/ connectivity results in PCS	Pertinent behavioral/ cognitive results in PCS
**High symptom (PCS+) studies**		
Amir 2021	• Reduced rsFC between right insula, lateral occipital cortex, and ACC<br>• Reduced rsFC in DMN<br>• Increased SN FC in right SMG (esp. right lat. occipital cortex and right sup. parietal lobule)<br>• Increased rsFC DLPFC with the right lat. occipital cortex<br>• Increased FC in sensorimotor and visual networks, DMN, and DAN, and reduced FC between visual network and right temporal occipital fusiform	• Significant negative correlation between the DAN rsFC values and behavioral symptoms of RPQ (irritability, depression, frustration, and restlessness) (p=0.05)<br>• Cognitive symptoms not correlated with any networks
Dumkrieger 2019	• Disrupted FC in pain and visual processing regions in PCS relative to HC and migraine groups	• Significant correlation between headache frequency with:<br>• static FC of left middle cingulate with right pulvinar and right posterior insula with left hypothalamus in the PPTH group<br>• dynamic FC in the PPTH group for one region pair (right middle cingulate with right supramarginal gyrus); nodes overlapping with SN
Leung 2016	• Reduced activation in sensory/pain processing regions (mostly SN nodes)<br>• Increased rsFC to inferior parietal lobe, premotor, and SSC2<br>• Granger causality revealed that ROIs (aligning with SN although not mentioned) receive significantly more inputs from other areas, whereas in HC these regions cast out more outward connections and receive much less inputs	• mTBI group demonstrated supraspinal activation particularly in regions associated with pain affective response (ACC), but not when exposed to a short period of noxious stimuli<br>• HC had robust responses from supraspinal modulatory regions (including the motor and PFCs) when exposed to the same intensity and duration of stimuli
Rockswold 2019	• Reduced FC between left lingual/parahippocampal seed and left middle frontal gyrus in the oculomotor dysfunction group; increased FC in the control group	• Significantly decreased fMRI responses in the visual areas during convergence task in the oculomotor dysfunction group compared with HC, correlating well to the visual symptoms of patients
Runyan 2022	• Reduced rsFC between left VLPFC, lateral premotor cortex, and DLPFC (nodes of frontoparietal network and dorsal and ventral attention networks) working memory regions and brain regions in the dorsal attention and somatomotor networks in both mTBI and PTSD groups versus controls<br>• Reduced FC between right DLPFC and left midbrain in mTBI compared to PTSD and control groups	• Only VLPFC connectivity (frontoparietal and ventral attention networks) was significantly associated with working memory performance across groups
Shafi 2020	• Hyperconnected frontal SN (left anterior insula with ACC and left right PFC; and left anterior insula with bilateral lateral PFC and lateral posterior parietal cortex)<br>• Hypoconnected parietal SN (right SMG with all 4 frontoparietal network nodes)<br>• No significant differences between PCS and HC groups in terms of DMN connectivity	• Nothing pertinent
Sheth 2021	• Enhanced FC between ACC and precuneus, occipital, somatosensory, and middle and posterior cingulate	• PTSD symptoms did not account for differences between groups

Supplemental Table 1 (continued): Pertinent descriptive results from included studies.

Study	Pertinent activation/ connectivity results in PCS	Pertinent behavioral/ cognitive results in PCS
Sours 2015	• Increased FC between DMN (PCC node) and TPN (dorsal ACC, bilateral insula, and left DLPFC) only significant in the chronic stage (compared to acute and subacute stages after mTBI)<br>• Reduced within network connectivity in DMN only present at chronic stage	• Despite normalized cognitive performance, chronic mTBI compared to HC demonstrates:<br>• increased rsFC between DMN and regions associated with SN and TPN<br>• reduced rsFC within DMN at the acute stage of injury
Stevens 2012	• Increased FC between precuneus and DMN<br>• Increased FC of left superior/inferior parietal lobules within the left frontoparietal network<br>• Reduced cingulate FC into DMN<br>• Reduced FC of motor regions with right SMG; sensorimotor cortex and caudate significantly less connected to the motor network; right globus pallidus and cerebellum FC correlated with PCS complaints<br>• Reduced FC of left middle frontal gyrus and superior/medial frontal gyri	• Total number of PCS symptoms was linearly associated with FC across multiple networks and brain regions, mainly ACC and SN, as well as medial frontal gyrus, inferior frontal gyrus, and precuneus
Trofimova 2021	• Increased FC between DMN and right middle frontal gyrus and post-central gyrus, as well as between vestibular-sensorimotor network and right PFC	• Vestibulo-oculo-motor symptoms correlated with increased rsFC in visual, vestibular, and multisensory processing networks
Vedaei 2023	• FC between right motor ventral and left superior temporal gyrus served as the most significant feature distinguishing mTBI/PCS+ from HC<br>• The following FCs were important features in the model prediction as well:<br>• between right ECN and right caudate<br>• between right SN and right middle temporal gyrus<br>• between DMN and left medial superior frontal cortex	• Nothing pertinent
Wong 2023	• Increased FC between DMN-SN<br>• Increased FC between both PCC and medial PFC seeds and left insula<br>• Increased FC between ECN and SN nodes (supplementary motor area and SMG)	• Increased FC between DMN (medial PFC seed) and right cerebellar lobes crus II and VII-b negatively correlated with scores on the graded symptom scale checklist<br>• Increased FC between ECN (left DLPFC seed) and subcortical SN nodes (right putamen and amygdala) positively correlated with cognitive scores<br>• Increased FC between ECN (right DLPFC seed) and DMN (medial PFC and left angular gyrus) positively correlated with cognitive scores

Supplemental Table 1 (continued): Pertinent descriptive results from included studies.

Study	Pertinent activation/ connectivity results in PCS	Pertinent behavioral/ cognitive results in PCS
**Low symptom (PCS-) studies**		
Chong 2019	• Reduced FC in the following regions at 1-month post-concussion: middle cingulate, posterior insula, middle occipital, spinal trigeminal nucleus, precentral (primary motor), and pulvinar<br>• Increased FC in middle cingulate, anterior and posterior insula, primary somatosensory area, spinal trigeminal nucleus, precentral, and pulvinar at 5-months versus 1-month post-concussion	• Significant negative correlation between somatosensory FC strengthening (1-month to 5-months post-concussion) and symptom severity at 5-months post-concussion
Churchill 2019	• Global FC had no significant effects at 1-year post-return to play	• Negative relationship between FC and clinical severity<br>• Stronger FC in patients recovering from PCS relative to HC
De Souza 2020	• Increased rsFC between DMN nodes (anterior medial PFC and PCC) and frontal and temporal regions (suggestive of recovery)	• No significant correlations between change scores on extended Glasgow outcome scale and connectivity between seed regions and significant peaks
D'Souza 2020	• Reduced FC in multiple networks, including the anterior DMN, ECN, somatomotor and auditory networks<br>• Increased FC in SMN and ECN for chronic versus acute mTBI	• Negative correlation between FC in SMN and symptom severity
McCuddy 2018	• Significant differences in most ROIs between 1-day and 1-month post-concussion were in SN	• Improvements in depressive symptoms over time correlated primarily with changes in connectivity between DMN and SN ("ventral attention")
**Studies that distinguished between high- versus low-symptom groups**		
Flowers 2021	• Reduced rsFC in (and greater imbalance between) pain modulation and sensory discrimination regions (right insula, bilateral premotor cortex, and left parietal)<br>• Increased FC in secondary somatosensory cortex	• Increase in headache severity in mTBI associated with imbalance in the supraspinal pain network, decline in supraspinal pain modulatory function, and enhancement of sensory/pain decoding
Sours 2013	• Reduction in anticorrelated networks for both with and without memory complaints for the DMN, but onlyd a reduction in the anticorrelated network for those with memory complaints for the TPN<br>• Increased FC with left inferior frontal gyrus and right superior parietal lobule for those with memory complaints; no regions of increased FC for those without memory complaints<br>• Increased FC with left superior temporal gyrus/insular cortex and left DLPFC for those with memory complaints in the mTBI group compared to HC<br>• Reduced FC with right angular gyrus for those without memory complaints in the mTBI group compared to HC	• Increased FC between TPN and SN associated with reduced memory performance<br>• Only PCS+ group had reduced anticorrelation of networks with the TPN, whereas both PCS+ and PCS- had reduced anticorrelation between other networks and DMN<br>• Increased FC between TPN and SN in PCS+ only, and patients with stronger correlation between these networks performed more poorly on memory tests

ACC: Anterior cingulate cortex. DAN: Dorsal attention network. DLPFC: Dorsolateral prefrontal cortex. DMN: Default mode network. ECN: Executive control network. FC: Functional connectivity. HC: Healthy controls. ICA: Independent component analyses. mTBI: Mild traumatic brain injury. PCC: Posterior cingulate cortex. PCS: Post-concussion syndrome. PFC: Prefrontal cortex. PPTH: Persistent post-traumatic headache. PTSD: Posttraumatic stress disorder. ROI: Region of interest. RPQ: Rivermead post-concussion symptoms questionnaire. Rs: Resting state. SSC2: Secondary somatosensory cortex. SMG: Supramarginal gyrus. SN: Salience network. TPN: Task-positive networks. VLPFC: Ventrolateral prefrontal cortex.

Supplemental Table 2: Neuroimaging details of included studies.

STUDY	Rs-fMRI Method	ROI selection (Networks)	WBA	Coord.	Statistical test reported	Scanner type	Total scan time	Source for coordinates	Contrast	# of Foci
**High Symptom (PSaC+) studies**										
Amir 2021	Mixed methods	a priori (DMN, SN, TPN)	Yes	MNI	t statistic	Siemens 3T	8 min	Table 2 & 3	PSaC+ > HC	12
Dumkrieger 2019	ROI-ROI	a priori (Visual)	No	MNI	Pearson's correlation	Siemens 3T	10 min	Figure 4 & 7	PSaC+ > HC	7
Leung 2016	ICA, SBC-voxel	a priori, evoked fMRI ("pain processing" network)	Yes	TAL	not reported	GE 1.5T	5 min	Table 4	PSaC+ > HC	10
Rockswold 2019	SBC-voxel	evoked fMRI (Visual)	Yes	MNI	Pearson's correlation	Siemens 3T	6 min 40s	Figure 2	PSaC+ > HC	1
Runyan 2022	SBC-voxel	a priori ("working memory network")	Yes	MNI	Z score	Siemens 3T	8 min	Table 2	PSaC+ > PTSD/OI	3
Shafi 2020	ROI-ROI	a priori (DMN, FPN, SN)	No	MNI	"Network based statistic"	SIGNA 3T	Not stated	Table 1, Figure 1	PSaC+ > HC	9
Sheth 2021	SBC-voxel	a priori (rostral ACC)	Yes	MNI	t statistic	Siemens 3T	8 min	Table 3 & 4	PSaC+ > HC	5
Sours 2015	ROI-ROI	a priori (DMN, TPN)	No	MNI	F statistic	Siemens 3T	5 min 42s	Table 2	PSaC+ > HC	8
Stevens 2012	ICA	ICA	Yes	MNI	t statistic	Siemens 3T	5 min 15s	Table 2	PSaC+ > HC	35
Trofimova 2017	Mixed methods	a priori ("vestibular" network)	Yes	MNI	t statistic	Siemens 3T	10 min	Figure 2 & 3, Table 2	PSaC+ > HC	9
Vedaei 2023	SBC-voxel	a priori, multi-network	Yes	MNI	Pearson's correlation, t statistic	Siemens 3T	6 min 10s	Table 4	PSaC+ > HC	55
Wong 2023	SBC-voxel	a priori (DMN, ECN)	Yes	MNI	t statistic	Siemens 3T	6 min 30s	Supplemental Table 1	PSaC+ > HC	11

Supplemental Table 2 (continued): Neuroimaging details of included studies.

STUDY	Rs-fMRI Method	ROI selection (Networks)	WBA	Coord.	Statistical test reported	Scanner type	Total scan time	Source for coordinates	Contrast	# of Foci
**Low symptom (PSaC-) studies**										
McCuddy 2018	ROI-ROI	a priori ("emotional processing" network)	No	MNI	t statistic	GE 3T	6 min	Figure 1	chronic > acute mTBI	27
D'Souza 2020	ICA	ICA	Yes	MNI	not reported	Siemens 3T	20 min	Table 5	chronic > acute mTBI	2
de Souza 2020	SBC-voxel	a priori (DMN)	Yes	MNI	not reported	Siemens 3T	not stated	Supplemental Table 1	chronic > acute mTBI	13
Chong 2019	ROI-ROI	a priori, evoked fMRI ("pain processing" network)	No	MNI	not reported	Siemens 3T	10 min	Table 1	chronic > acute mTBI	7
Churchill 2019	Gconn	global connectivity	Yes	MNI	Pearson's correlation, t statistic	Siemens 3T	not stated	Table 4	chronic > acute mTBI	5
**Studies that distinguished between high- versus low-symptom groups**										
Sours 2013	SBC-voxel	a priori (DMN, SN, TPN)	Yes	MNI	F statistic	Siemens 3T	5 min 42s	Supplemental Table 1 & 2	PSaC+ > PSaC-	18 (7 PSaC+, 11 PSaC-)
Flowers 2021	ICA	ICA (“pain processing regions”)	Yes	TAL	t statistic	GE 3T	6 min	Figure 2	PSaC+ > PSaC-	8 (6 PSaC+, 2 PSaC-)

DMN: default mode network. ECN: executive control network. HC: healthy control. ICA: independent component analysis. MNI: Montreal Neurologic Institute. OI: orthopedic injury control. PSaC: post-concussive syndrome. PTSD: posttraumatic stress disorder. ROI: region of interest. SBC: seed-based connectivity. SN: salience network. TAL: Talairach. TPN: task positive network. WBA: whole brain analysis.

Supplementary Table 3: Additional characteristics of included studies.

Study	Moderate-severe TBI excluded	Psychiatric conditions excluded	History of previous concussion excluded
**High symptom (PSaC+) studies**			
Amir 2021[70]	Yes	Yes	Yes
Dumkrieger 2019[61]	Yes	No	No
Leung 2016[60]	Yes	Yes	No
Rockswold 2019[64]	Yes	No	No
Runyan 2022[66]	Yes	Yes	Yes
Shafi 2020[10]	Yes	No	No
Sheth 2021[67]	Yes	No	No
Sours 2015[69]	Yes	Yes	Not stated
Stevens 2012[68]	Yes	Yes	Yes
Trofimova 2021[63]	Yes	Not stated	No
Vedaei 2023[72]	Yes	Yes	No
Wong 2023[71]	Yes	No	No
**Low symptom (PSaC-) studies**			
Chong 2019[78]	Yes	Not stated	No
Churchill 2019[6]	Yes	No	No
De Souza 2020[98]	No*	Not stated	Not stated
D'Souza 2020[99]	Yes	Yes	Yes
McCuddy 2018[97]	Yes	Yes	No
**Studies that distinguished between high- versus low-symptom groups**			
Flowers 2021[62]	Yes	Yes	Yes
Sours 2013[65]	Yes	Yes	Yes

*Included 2 moderate severity TBI patients

Supplementary Table 4: Scores for PSaC symptom burden thresholds (for applicable studies)

Study	Primary clinical outcome measure	Score (mean)	Threshold score suggested in available literature
**High symptom (PSaC+) studies**			
Stevens 2012	PCSC[112]	6	6
Trofimova 2021	PCSS[113]	43.7	7
	VOMS[114]	2.9	2
Wong 2023	GSSC[115]	41	12
**Low symptom (PSaC-) studies**†			
Chong 2019	SCAT-3[116,117]	21*	>3
Churchill 2019	SCAT-3[116,117]	1	>3
De Souza 2020	GOS-E[118]	7	Categorical**
D'Souza 2020	RPQ[119]	7.1	>14
McCuddy 2018	HAM-D[120]	3.5	>8

† Scores for PSaC– studies reflect the chronic/recovery phase at follow-up, rather than acute injury.
* This study showed a ~50% reduction in severity over five months, reflecting progressive symptom resolution. Despite residual symptoms, its recovery trajectory supports classification within the low symptom/recovery (PSaC–) group.
**Categorical scale from 1 (death) to 8 (full recovery) whereby 7 indicates "minor physical or mental deficits but able to resume normal life".

GSSC: Graded Symptom Scale Checklist. GOSE: Glasgow Outcome Scale-Extended. PCSC: Post-Concussion Symptom Checklist. PCSS: Post-Concussion Symptom Scale. RPQ: Rivermead Post-Concussion Questionnaire. VOMS: Vestibular/Ocular Motor Screening.

References

112. Sawchyn, J. M., Brulot, M. M. & Strauss, E. Note on the Use of the Postconcussion Syndrome Checklist. *Arch. Clin. Neuropsychol.* **15**, 1–8 (2000).

113. Eagle, S. R. *et al.* Concussion Symptom Cutoffs for Identification and Prognosis of Sports-Related Concussion: Role of Time Since Injury. *Am. J. Sports Med.* **48**, 2544–2551 (2020).

114. Kontos, A. P. *et al.* Discriminative Validity of Vestibular Ocular Motor Screening in Identifying Concussion Among Collegiate Athletes: A National Collegiate Athletic Association–Department of Defense Concussion Assessment, Research, and Education Consortium Study. *Am. J. Sports Med.* **49**, 2211–2217 (2021).

115. Grubenhoff, J. A., Kirkwood, M., Gao, D., Deakyne, S. & Wathen, J. Evaluation of the Standardized Assessment of Concussion in a Pediatric Emergency Department. *Pediatrics* **126**, 688–695 (2010).

116. Chin, E. Y., Nelson, L. D., Barr, W. B., McCrory, P. & McCrea, M. A. Reliability and Validity of the Sport Concussion Assessment Tool–3 (SCAT3) in High School and Collegiate Athletes. *Am. J. Sports Med.* **44**, 2276–2285 (2016).

117. Downey, R. I., Hutchison, M. G. & Comper, P. Determining sensitivity and specificity of the Sport Concussion Assessment Tool 3 (SCAT3) components in university athletes. *Brain Inj.* **32**, 1345–1352 (2018).

118. Mitra, B. *et al.* Long-term outcomes of major trauma patients with concussion. *Injury* **54**, 75–81 (2023).

119. Zeldovich, M. *et al.* Reference Values for the Rivermead Post-Concussion Symptoms Questionnaire (RPQ) from General Population Samples in the United Kingdom, Italy, and The Netherlands. *J. Clin. Med.* **11**, 4658 (2022).

120. Zimmerman, M., Martinez, J. H., Young, D., Chelminski, I. & Dalrymple, K. Severity classification on the Hamilton depression rating scale. *J. Affect. Disord.* **150**, 384–388 (2013).

Supplemental Table 5: Quality control assessment of included studies.

Study	fMRI quality control	Adequately powered	Multi- versus single-center study	Research question clearly stated	Study population clearly defined	Participation of eligible persons at least 50%	Consistent recruitment in well-defined period	Inclusion/ exclusion criteria uniformly applied	Sample size justification/ power description provided	Loss to follow-up less than 20%
**High symptom (PCS+) studies**										
Amir 2021	Yes	Not stated	Single	Yes	Yes	Yes	Yes	Yes	No	N/A
Dumkrieger 2019	Yes	Not stated	Multi	Yes	Yes	Yes	Yes	Yes	No	Yes
Leung 2016	Yes	Not stated	Single	Yes	Yes	Yes	Yes	Yes	No	N/A
Rockswold 2019	Yes	Not stated	Single	Yes	Yes	Yes	Not stated	Yes	No	Yes
Runyan 2022	Yes	Not stated	Single	Yes	Yes	Yes	Yes	Yes	No	N/A
Shafi 2020	Yes	Not stated	Single	Yes	Yes	Yes	Yes	Yes	No	N/A
Sheth 2021	Yes	Not stated	Multi	Yes	Yes	Not stated	Not stated	Yes	No	N/A
Sours 2015	Yes	Not stated	Single	Yes	Yes	Yes	Yes	Yes	No	Yes
Stevens 2012	Yes	Not stated	Single	Yes	Yes	Yes	Not stated	Yes	No	N/A
Trofimova 2021	Yes	Not stated	Single	Yes	Yes	Yes	Yes	Yes	Yes	N/A
Vedaei 2023	Yes	Yes	Single	Yes	Yes	Yes	Yes	Yes	Yes	Yes
Wong 2023	Yes	Not stated	Single	Yes	Yes	Yes	Yes	Yes	No	N/A
**Low symptom (PCS-) studies**										
Chong 2019	Yes	Not stated	Single	Yes	Yes	Yes	Yes	Yes	No	Yes
Churchill 2019	Yes	Not stated	Single	Yes	Yes	Yes	Yes	No	No	No
De Souza 2020	Yes	Not stated	Single	Yes	No	Yes	Yes	No	No	Yes
D'Souza 2020	Yes	Not stated	Single	Yes	Yes	No	Yes	Yes	No	No
McCuddy 2018	Yes	Yes	Single	Yes	Yes	Yes	Yes	Yes	No	Yes
**Studies that distinguished between high- versus low-symptom groups**										
Flowers 2021	Yes	Not stated	Single	Yes	Yes	Not stated	Not stated	Yes	No	N/A
Sours 2013	Yes	Not stated	Single	Yes	Yes	Yes	Yes	Yes	No	N/A

fMRI: Functional magnetic resonance imaging. N/A: Not applicable.

Supplemental Table 6: Results of Quality Assessment Tool for Observational Cohort and Cross-Sectional Studies for included studies.

Study	Amir 2021	Dumk. 2019	Leun. 2016	Rocks. 2019	Runy. 2022	Shafi 2020	Sheth 2021	Sour. 2015	Steve. 2012	Trofim. 2021	Veda. 2023	Wong 2023	Chong 2019	Church. 2019	deSou. 2020	D'Souz. 2020	McCuddy 2018	Flowers 2021	Sours 2013
**Overall Score**	10/11	5/11	8/11	10/11	6/11	10/11	7/11	4/11	11/12	12/13	10/11	9/11	12/13	9/11	11/13	10/13	7/13	9/13	11/13
**Overall Quality**	Good	Poor	Good	Good	Fair	Good	Good	Poor	Good	Good	Good	Good	Good	Good	Good	Good	Fair	Good	Good

Supplementary Table 7: Summary of total number of connections between the salience network and other large-scale networks based on extracted MNI coordinates from PCS+ versus PCS- studies.

Networks	Number of connections		Percentage of total connections	
	PCS+	PCS-	PCS+	PCS-
SN-ECN	10	1	17%	3%
SN-DAN	3	0	5%	0%
SN-DMN	19	6	33%	35%
SN-SMN	5	0	8%	0%
SN-PVN	6	2	10%	12%
SN-LMB	3	2	4%	12%

SN = salience network, DMN = default mode network, ECN = executive control network, DAN = dorsal attention network, SMN = somatosensory network, PVN = primary visual network, LMB = limbic network

Supplemental Table 8: Neurosynth term study information

Term	Number of studies	Included neuropsychiatric disorders
Depressive Disorders	218	Mood disorders
Anxiety Disorders	95	Anxiety disorders
Angry	159	Mostly healthy subjects; **chronic TBI (1 study),** spinal cord injury (1 study), impulsive disorder (2 studies)
Impulsivity	120	Mostly healthy subjects; ADHD (19 studies), substance use disorders (12 studies), borderline personality disorder (7 studies), mood disorder (3 studies)
Sleep	154	Mostly healthy subjects; obstructive sleep apnea (9 studies), anxiety disorder mood disorder, PTSD (1 study each)
Pain	516	Various pain conditions (e.g., fibromyalgia, chronic back pain, neuropathic pain, etc)
Arousal	295	Mostly healthy subjects; anxiety disorder (9 studies), mood disorder (5 studies), anxiety disorder (4 studies), PTSD (4 studies), brain injury (2 studies)
Concentration	81	Mostly healthy subjects; substance use disoders (3 studies), mood disorder (2 studies, anxiety disorder (2 studies), **TBI (1 study)**
Memory	2744	Mostly healthy subjects; schizophrenia (129 studies), mood disorders (50 studies), mild cognitive impairment (40 studies), dementia (30 studies), substance use disorders (13 studies), anxiety disorders (12 studies), stroke (3 studies), **TBI (19 studies)**
Auditory stimuli	115	Mostly healthy subjects; mood disorder (2 studies)
Visual stimuli	270	Mostly healthy subjects; mood disorder (2 studies), anxiety disorder (2 studies)
Concussion*	37	**Concussion (37 studies)**

*Using the *Neurosynth Compose* tool

Supplementary Methods Section: Network-Based Meta-Analysis

Concatenation of the two different phase-encoded data (right to left, left to right) ensured that any potential (but likely trivial) effect of phase encoding on gradient direction was counterbalanced by the opposing phase encoding.[121] Importantly, aside from the direction of acquisition (right to left, left to right), scanning parameters were identical across sessions, and these sessions were performed on the same day.[122] Concatenation improves the reproducibility of resting state functional connectivity (FC) measures, as the test–retest reliability of these measures scales upward with increasing data and number of sessions.[123-127]

Acquired images were preprocessed by the Human Connectome Project (HCP) according to the HCP functional preprocessing pipeline, which involves: (i) spatial and gradient distortion corrections; (ii) correction of head movement; (iii) intensity normalization; (iv) single spline re-sampling of EPI frames into 2mm isotropic MNI space; and (v) HCP's FIX+ICA pipeline for the removal of temporal artefacts. Refer to Glasser et al.[128] and Smith et al.[122] for further details on HCP resting-state functional MRI acquisition and preprocessing. In addition to the HCP minimal preprocessing, band pass temporal filtering (BPTF; 0.01-0.1Hz) was employed. Analyses were performed on the data with minimal spatial smoothing (4 mm full width at half maximum, FWHM) to reduce loss of spatial information and spurious shifts in boundaries between gray and white matter.[129]

References:

121. Tian, Y., Margulies, D. S., Breakspear, M. & Zalesky, A. Topographic organization of the human subcortex unveiled with functional connectivity gradients. Nat. Neurosci 2020. 23, 1421–1432

122. Smith, S. M. et al. Resting-state fMRI in the Human Connectome Project. Neuroimage 2013. 80, 144–168

123. Birn, R. M. et al. The effect of scan length on the reliability of resting-state fMRI connectivity estimates. Neuroimage 2013;83, 550–558.

124. Noble, S. et al. Influences on the test–retest reliability of functional connectivity MRI and its relationship with behavioral utility. Cerebral Cortex 2017;27, 5415–5429.

125. Choe, A. S. et al. Reproducibility and temporal structure in weekly resting-state fMRI over a period of 3.5 years. PLoS ONE 2015;10, e0140134.

126. Gordon, E. M. et al. Precision functional mapping of individual human brains. Neuron 2017; 95, 791–807.e7.

127. Cui, Z. et al. Individual variation in functional topography of association networks in youth. Neuron 2020;106, 340–353.e8.

128. Glasser, M. F. et al. The minimal preprocessing pipelines for the Human Connectome Project. Neuroimage 2013;80, 105–124

129. Coalson, T. S., Van Essen, D. C. & Glasser, M. F. The impact of traditional neuroimaging methods on the spatial localization of cortical areas. Proc. Natl Acad. Sci. 2018; USA 115, E6356–E6365.
